# Single-cell-resolved dynamics of chromatin architecture delineate cell and regulatory states in zebrafish embryos

**DOI:** 10.1016/j.xgen.2021.100083

**Published:** 2022-01-13

**Authors:** Alison C. McGarvey, Wolfgang Kopp, Dubravka Vučićević, Kenny Mattonet, Rieke Kempfer, Antje Hirsekorn, Ilija Bilić, Marine Gil, Alexandra Trinks, Anne Margarete Merks, Daniela Panáková, Ana Pombo, Altuna Akalin, Jan Philipp Junker, Didier Y.R. Stainier, David Garfield, Uwe Ohler, Scott Allen Lacadie

**Affiliations:** 1 Computational Regulatory Genomics, Berlin Institute for Medical Systems Biology (BIMSB), Max Delbrück Center for Molecular Medicine, Berlin 10115, Germany; 2 Quantitative Developmental Biology, Berlin Institute for Medical Systems Biology, Max Delbrück Center for Molecular Medicine, Berlin 10115, Germany; 3 Bioinformatics and Omics Data Science Platform, Berlin Institute for Medical Systems Biology, Max Delbrück Centre for Molecular Medicine, Berlin 10115, Germany; 4 Department of Developmental Genetics, Max Planck Institute for Heart and Lung Research, Bad Nauheim 61231, Germany; 5 Epigenetic Regulation and Chromatin Architecture, Berlin Institute for Medical Systems Biology, Max Delbrück Centre for Molecular Medicine, Berlin, Germany; 6 Institute for Biology, Humboldt Universität Berlin, Berlin 10115, Germany; 7 IRI Life Sciences, Humboldt Universität Berlin, Berlin 10115, Germany; 8 Electrochemical Signaling in Development and Disease, Max Delbrück Centre for Molecular Medicine, Berlin, Germany; 9 DZHK (German Centre for Cardiovascular Research), partner site Berlin, Berlin 13125, Germany; 10 Berlin Institute of Health, Berlin 10178, Germany

**Keywords:** zebrafish, chromatin, cis regulatory element, enhancer, promoter, single-cell ATAC-seq, Hi-C, npas4l

## Abstract

DNA accessibility of *cis*-regulatory elements (CREs) dictates transcriptional activity and drives cell differentiation during development. While many genes regulating embryonic development have been identified, the underlying CRE dynamics controlling their expression remain largely uncharacterized. To address this, we produced a multimodal resource and genomic regulatory map for the zebrafish community, which integrates single-cell combinatorial indexing assay for transposase-accessible chromatin with high-throughput sequencing (sci-ATAC-seq) with bulk histone PTMs and Hi-C data to achieve a genome-wide classification of the regulatory architecture determining transcriptional activity in the 24-h post-fertilization (hpf) embryo. We characterized the genome-wide chromatin architecture at bulk and single-cell resolution, applying sci-ATAC-seq on whole 24-hpf stage zebrafish embryos, generating accessibility profiles for ∼23,000 single nuclei. We developed a genome segmentation method, ScregSeg (single-cell regulatory landscape segmentation), for defining regulatory programs, and candidate CREs, specific to one or more cell types. We integrated the ScregSeg output with bulk measurements for histone post-translational modifications and 3D genome organization and identified new regulatory principles between chromatin modalities prevalent during zebrafish development. Sci-ATAC-seq profiling of *npas4l*/*cloche* mutant embryos identified novel cellular roles for this hematovascular transcriptional master regulator and suggests an intricate mechanism regulating its expression. Our work defines regulatory architecture and principles in the zebrafish embryo and establishes a resource of cell-type-specific genome-wide regulatory annotations and candidate CREs, providing a valuable open resource for genomics, developmental, molecular, and computational biology.

## Highlights

• Single-cell DNA accessibility defines 17 cell types from the 24-hpf zebrafish embryo
• Machine learning with ScregSeg classifies complex regulatory programs genome-wide
• Integrating sc and bulk chromatin data reveals cross-modal regulatory principles
• sci-ATAC profiling of npas4l mutants exposes unexpected changes in cell composition

## Graphical abstract

**Figure:**
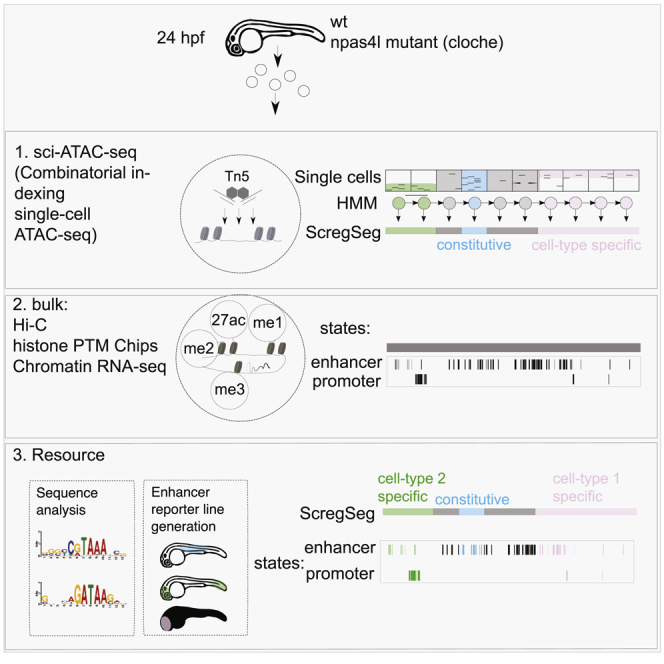

## Teaser

Accessibility measurements at single-cell resolution define cell states in the highly heterogeneous zebrafish embryo and reveal unexpected cellular phenotypes in genetic mutants. Genome-wide classification of complex cross-cell-type regulatory programs using machine learning provides a solid resource for future studies with immediate direct effects on transgenic reporter gene design, candidate identification for perturbation studies, and regulatory sequence annotation for the advancement of predictive models. Integrating single-cell data with bulk chromatin measurements reveals principled relationships between transcriptional regulatory mechanisms.

## Introduction

The coordination of *cis*-regulatory elements (CREs) is essential to the tight regulation of gene expression programs that direct cell fate changes in embryonic development. The types of CREs include promoters, enhancers, insulators, and silencers, whose sequence and dynamic physical properties determine their function. The fundamental unit of a CRE is a nucleosome-depleted region (NDR), which acts as a binding platform for transcriptional regulators and can be highly dynamic across cell types due to the combined action of pioneering factors and nucleosome remodelers. Mammalian NDRs often harbor divergently oriented core promoter sequence elements and transcription start sites (TSSs) and are flanked by well-positioned nucleosomes whose histone post-translational modifications (PTMs) reflect the activation state and/or class of CRE.^1^^,^^2^ This complex architecture has been described as the regulatory interface between the genome and its functional output.^3^

The development of single-cell high-throughput molecular assays^4^, ^5^, ^6^ has revolutionized systems genomics, allowing the extensive profiling of cell-type diversity of almost any tissue or organism with little to no prior information. The assay for transposase-accessible chromatin using sequencing^7^ can quantify the extent of CRE nucleosome depletion on a genome-wide scale. Its further development has enabled the measure of chromatin accessibility in tens of thousands of single cells from such diverse biological contexts as cell lines,^8^^,^^9^ *Drosophila* embryos,^10^ primary tissues,^11^, ^12^, ^13^, ^14^, ^15^, ^16^ and human organoids.^17^ These datasets have generated comprehensive resources of putative distal regulatory elements, transcriptional regulators, cell-type specificity of inherited disease-associated traits,^11^^,^^16^ putative higher-order interactions between regulatory elements,^9^ and epigenomic contribution to lineage priming.^15^ However, single-cell assay for transposase-accessible chromatin with high-throughput sequencing (scATAC-seq) data present several distinct analysis challenges from single-cell RNA-sequencing (scRNA-seq) measurements, such as higher sparsity and feature dimensionality, as well as typically unknown input regions.^18^ Therefore, computational method development for this data type is an important and ongoing effort.

The zebrafish has a long history as a model system for embryology, and forward genetic screens have identified many genes with key roles during vertebrate development.^19^ Zebrafish have been used increasingly for cutting-edge genomic profiling,^20^, ^21^, ^22^, ^23^, ^24^, ^25^ but its *cis*-regulatory dynamics have yet to be characterized at single-cell resolution. Furthermore, key resources available for mouse or human genomics studies, such as genome classifications based on histone PTM chromatin immunoprecipitation sequencing (ChIP-seq) signals, high-depth genome-wide probing of three-dimensional (3D) chromatin spatial organization, and databases of regulatory elements are limited for the zebrafish community.

In this study, we characterized the genome-wide chromatin architecture of the whole 24-h post-fertilization (hpf)-stage zebrafish embryo, at bulk and single-cell resolution, to generate a resource of cell-type-specific candidate CREs. We applied single-cell combinatorial indexing ATAC-seq (sci-ATAC-seq)^10^ to whole embryos, generating accessibility profiles for ∼23,000 single nuclei. Taking inspiration from chromatin segmentation,^26^, ^27^, ^28^ we developed a hidden Markov model (HMM)-based algorithm called single-cell regulatory landscape segmentation, or ScregSeg, to classify regions of the genome into a number of distinct states based on either single-cell or cell-group-collapsed (pseudo-bulk) accessibility tracks. We use this approach (1) to select initial informative genomic regions for subsequent dimensionality reduction and cell clustering, and (2) for an unbiased characterization of complex combinatorial cell-specific CRE dynamics that go beyond and are independent of the typical peak calling and differential accessibility analysis. We show that diverse cell types present in the 24-hpf embryo can be identified by their accessibility profiles and have identified complex patterns of CRE dynamics that reflect the combinatorial nature of transcriptional regulation. Sequence analysis of these *cis*-regulators allows us to infer putative transcription factors (TFs) that bind chromatin in a cell-type-specific manner. Using bulk ChIP-seq data for histone PTMs known to occur at CREs, we provide the additional resource of a genome-wide classification for promoter- and enhancer-like chromatin states at the 24-hpf stage. Integrating these classifications with sci-ATAC-seq and bulk *in situ* Hi-C, we show clear relationships between promoter-like states, constitutive accessibility, and 3D insulation, as well as between co-accessibility and 3D interactions, thereby expanding insight into regulatory principles that are active during zebrafish development. Lastly, we apply sci-ATAC-seq to embryos harboring a mutation in the *cloche* gene *npas4l*, which lack blood and endothelial cells,^29^^,^^30^ and observe hitherto undescribed changes in muscle and epidermal cell numbers. We detect and validate candidate cell-type-specific CREs around the *npas4l* locus, suggesting an intricate network upstream of this hematovascular transcriptional master regulator. We provide intuitive access to our data and analyses via an interactive browser (https://scbrowse.mdc-berlin.de/) and the University of California, Santa Cruz (UCSC) genome browser hub (http://genome.ucsc.edu/cgi-bin/hgTracks?db=danRer11&hubUrl=https://bimsbstatic.mdc-berlin.de/hubs/ohler/scipipe_v4/hub.txt).

## Results

### Single-nucleus accessibility profiles separate whole embryos into cell types

**Figure 1:**
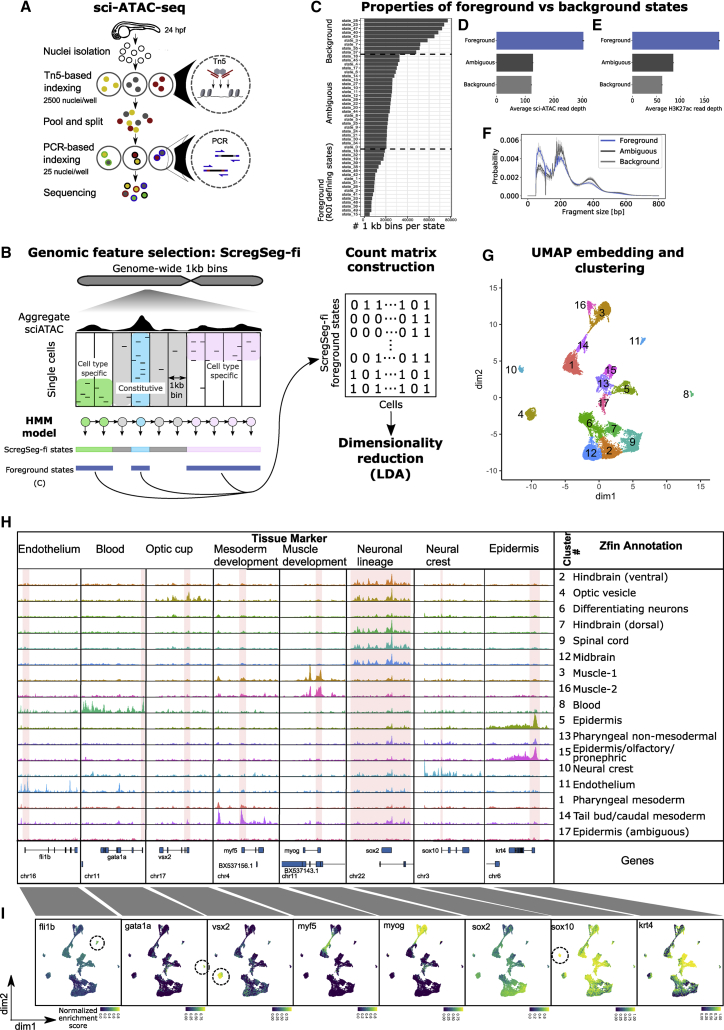
Generating cell-type-specific accessibility profiles from 24-hpf zebrafish embryos (A) Schematic of sci-ATAC-seq method. Nuclei are extracted from flash-frozen whole embryos staged at 24 hpf. Nuclei are sorted into 96-well plates, 2,500 per well, and barcoded during tagmentation. Tagmented nuclei are pooled and then split into 96-well plates, 25 per well, and a second set of barcodes introduced by PCR. The resulting DNA fragments are pooled and sequenced, with unique barcode combinations representing single cells. (B) Schematic representation of ScregSeg for genomic feature identification (ScregSeg-fi). The genome is divided into 1-kb bins and segmented using an HMM that assigns a state to each bin based on the accessibility distribution over cells. Subsequently, putative informative (foreground) states are used to define the regions of interest for the dimensionality reduction using latent Dirichlet allocation (LDA). (C) Number of 1-kb regions per state (state frequency). States with low, medium, or high numbers of assigned genomic regions were grouped into “foreground,” “ambiguous,” and “background.” Foreground states were selected based on the state frequency. (D) Average number of sci-ATAC-seq reads at 1-kb genomic regions assigned to foreground, ambiguous, and background states. Error bars indicate the 95% confidence intervals around the mean (as determined by seaborn.barplot). (E) Average number of bulk H3K27sc ChIP-seq reads at 1-kb genomic regions assigned to foreground, background, or ambiguous states. Error bars indicate the 95% confidence intervals around the mean (as determined by seaborn.barplot). (F) Normalized fragment size distribution of 1-kb genomic regions assigned to foreground, ambiguous, and background states. Ribbons represent 95% confidence intervals around the mean (as determined by seaborn.lineplot). (G) UMAP representation of the dimensionality-reduced and batch-corrected feature matrix (cell-Topic matrix) on ∼23,000 cells. Colors represent the 17 clusters determined by density clustering. (H) Summary pseudo-bulk chromatin accessibility profiles from aggregated cells for each density cluster at marker genes of major tissues and cell types of 24-hpf zebrafish embryos. Consensus annotations derived from enrichment of genes that map to differentially accessible segments per cluster, with ZFIN anatomical database terms and published cell-type markers.^25^ (I) Per-cell distribution of accessibility at regions covering the promoters of marker genes, represented in UMAP space. Color represents the rank-based AUCell enrichment score for a given region.^35^^,^^36^

We set out to determine a comprehensive genomic regulatory map of the zebrafish, *Danio rerio*, at the 24-hpf stage with the bipartite goal of (1) uncovering regulatory architecture and principles and (2) establishing a resource of genome-wide regulatory annotations for future studies. At 24 hpf, zebrafish embryos have established the classic bilateral vertebrate body plan and are at a key transitional point of cell-type specification and organogenesis, arguably the most morphologically comparable across diverse vertebrate embryos.^31^^,^^32^ DNA accessibility within chromatin is highly variable between cell types,^33^ mostly reflecting regulatory differences. We, therefore, used single-nucleus combinatorial indexing^10^ to determine genome-wide accessibility profiles for many cell types in parallel from whole zebrafish embryos. Nuclei were isolated from staged embryos, subjected to two rounds of barcoding via tagmentation and PCR with random mixing in between, and the resulting DNA fragments sequenced to high depth (Figure 1A). Species mixing with nuclei from the sea urchin demonstrated single-cell resolution with a barcode-collision rate of ∼14%–15%,^34^ and a distinct distribution of barcodes with >1,000 unique reads was considered to represent intact nuclei (Figures S1A and S1B). In all, we sequenced ∼23,000 nuclei with an average depth per nucleus of >10,000 unique fragments from 3 independent experiments, with 24 hpf embryos from wild-type (two experiments) or *npas4l* (*cloche*) mutant lines (see below for detailed characterization).

Whereas single-cell RNA expression measurements are typically quantified based on known gene annotations, the initial definition of genomic regions of interest (also referred to as features) to be quantified from single-nucleus accessibility maps poses a challenge. Current solutions to this issue aggregate the data from all cells to identify highly accessible regions that may subsequently be refined through an iteration of clustering, cell aggregation within clusters, and re-selection of regions, as in iterative latent semantic indexing (LSI).^15^^,^^37^ Here, we propose an alternative strategy to identify relevant regions that represent the chromatin accessibility of all captured cell types using an HMM. The HMM takes the observed accessibility for each genomic region in each single nucleus and summarizes the distinct cross-cell accessibility profiles (referred to as states) that underlie these observations while accounting for correlations between neighboring genomic regions. Specifically, we use a 50-state HMM with Dirichlet multinomial emission probabilities and tracks for each single nucleus at 1-kb resolution. The model is then used to infer the most probable state for each 1-kb region across the genome. We observe that some states (accessibility profiles) represent accessibility in all cells (constitutive), while others show high cell-type-specific accessibility, or minimal, sporadic accessibility, likely constituting genomic background signals (Figures 1B and S1C). To establish informative (foreground) regions for the downstream analysis, we selected those confidently associated with HMM states that cover <1.5% of the genome, resulting in 71,550 features after further processing (Figure 1C; see STAR Methods). The likely functional relevance of these regions is supported by on average (1) higher read coverage across the cells, (2) higher ChIP-seq signal for the CRE-associated histone PTM H3K27ac, (3) enrichment for short fragments associated with NDRs, and (4) the tendency to be enriched among accessible regions specific to subpopulations of cells (Figures 1C–1F and S1C). Foreground state regions are then used as features for the downstream dimensionality reduction step. We refer to this approach as single-cell regulatory landscape segmentation for feature identification (ScregSeg-fi). ScregSeg-fi analyses are scalable to large datasets through memory-efficient and parallelized processing (see Note S1). Benchmarking feature selection by ScregSeg-fi on several external scATAC-seq datasets, alongside our zebrafish sci-ATAC-seq, demonstrated that it leads to comparable or improved clustering performance relative to feature selection by iterative LSI (Figures S1D and S1E; see Note S1 for a detailed comparison). After dimensionality reduction with latent Dirichlet allocation (LDA) using cisTopic^35^ (Figures 1B and S1F), the resulting low-dimensional matrix was subjected to batch correction with a linear regression model, followed by uniform manifold approximation and projection (UMAP) transformation (Figure S1G; see STAR Methods). Finally, grouping nuclei according to density in the UMAP space led to 17 clusters as candidate cell types (Figure 1G; see STAR Methods).

To annotate the sci-ATAC-seq clusters, we tested whether cluster-specific differentially accessible regions are enriched in the vicinity of gene sets defined by anatomical features from the ZFIN database or cluster-specific marker genes from a published stage-matched scRNA-seq dataset^25^^,^^38^ and confirmed the cell-type annotations through visual inspection of known cell-type marker genes (Figures 1H and 1I; Tables S1 and S2; see STAR Methods). Endothelium, blood, neural crest, and optic vesicle annotations could be confidently assigned to four distinct clusters (Figure 1G, clusters 11, 8, 10, and 4, respectively), showing accessibility around known marker genes *fli1b*,^39^ *gata1a*,^40^^,^^41^ *sox10*,^42^ and *vsx2*,^43^ respectively (Figures 1H and 1I). Three separate territories with substructure were also observed:

(1) A largely mesodermal territory encompassing clusters 16 and 3, which show high accessibility around muscle cell marker genes such as *myog*; cluster 14, which has high accessibility around early mesoderm markers such as *myf5,* presumably representing less differentiated tailbud precursors and caudal mesoderm; and cluster 1, which is enriched with pharyngeal mesoderm markers. The division of muscle into clusters 16 and 3 is possibly due to the remaining local batch effects (Figure S1G; see STAR Methods).
(2) In the center of the map, clusters 5 and 15 have high accessibility around epidermal and peridermal markers such as *krt4*, and in cluster 15, “olfactory placode” and “pronephric duct” annotations; cluster 13 is enriched for “pharyngeal arch” and “skeletal” terms, and given its location in the UMAP space, is likely to represent non-mesodermal contributions to the pharyngeal arches; cluster 17, which, despite showing low but significant enrichment for epidermal terms, displays a relatively flat accessibility profile, suggesting it may represent a different cell state such as mitotic or dying cells, although no such Gene Ontology (GO) terms were enriched (data not shown).
(3) A largely neuronal territory with broad enrichment around the early neuronal regulator *sox2*^44^ that is subdivided into cluster 9, representing spinal cord; clusters 7 and 2, representing hindbrain; cluster 6, representing differentiating neurons; and cluster 12, representing midbrain (Figures 1G–1I; Table S1). Based on these marker gene associations, the clusters are, from this point on, assigned representative names (Figure 1H).

### ScregSeg defines single- and multi-cluster-specific accessibility dynamics

**Figure 2:**
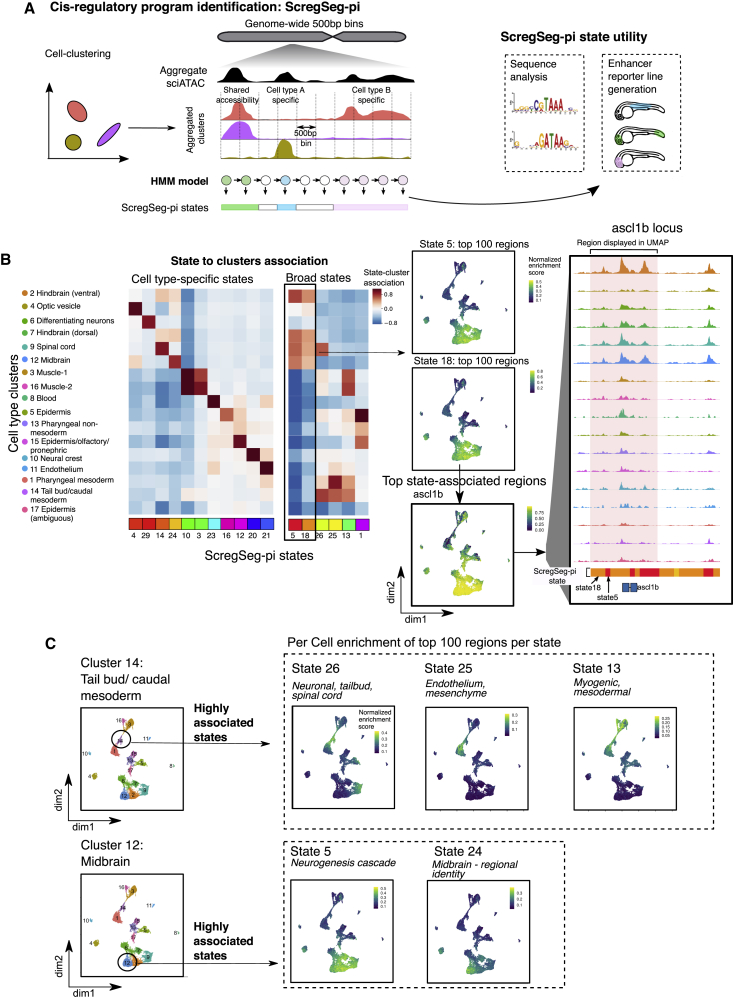
Segmentation of accessibility profiles reveals cell-type-specific and shared regulatory regions (A) Schematic representation of ScregSeg for identifying regulatory programs (ScregSeg-pi). The genome is divided into 500-bp bins and segmented based on the accessibility distribution over the cluster-collapsed accessibility tracks using a 30-state HMM. The bins are assigned to states that give rise to the classification of the regulatory landscape of the genome. (B) Heatmap representing the association between states and clusters based on the log-ratio between the states’ emission probabilities and the (normalized) overall read coverage per cluster, which accounts for read depth per cluster (see STAR Methods). Display restricted to states showing the strongest association with single-cell-type clusters (left) and multiple-cell-type clusters (right; full heatmap in Figure S2A). ScregSeg-pi states 5 and 18 (highlighted) encompass CREs accessible in 4 neuronal cell-type clusters. Accessibility of 100 CREs with the highest assignment probability for these states represented in UMAP space, in which the color represents the rank-based AUCell enrichment score for a given region.^35^^,^^36^ Loci around *ascl1b* have a high probability of assignment to states 5 and 18, as indicated below the cluster-aggregated accessibility tracks, and the *ascl1b* locus (highlighted in pink) shows a high normalized enrichment in the UMAP territory representing neuronal cell types. (C) Two examples of a cell-type cluster (tail bud and midbrain) that are associated with multiple ScregSeg-pi states (26, 25, and 13 and 5 and 24, respectively). The right-hand panel shows the per-cell distribution of accessibility at the gene body of genes mapping to segments, with the top 100 logFC enrichments for each of these states.

Cell diversity results from the implementation of regulatory “programs,” which represent unique combinatorial activities of both *cis* and *trans* regulators. Importantly, individual components of these programs may be reused in several different contexts. A typical differential accessibility analysis selects regions that are significantly attributed to a predefined set of foreground cells compared to a selected set of background cells. This introduces a bias against complex multi-cell-type accessibility patterns and could lead to a false assignment to a single cell type only. We reasoned that an HMM facilitates unbiased characterization of the regulatory landscape, as it does not require pre-definition of foreground and background cell types. Therefore, we applied ScregSeg again, this time on cluster-collapsed accessibility profiles (i.e., with an input track for each of the 17 cell types defined in Figure 1 instead of single cells). We used 30 states to characterize the genome with 500-bp resolution (Figures 2A and S2A–S2C). The state representation of the HMM identifies individual regulatory programs such as accessibility specific to a single or several clusters, as well as background accessibility (Figures 2B and S2A; discussed in further detail below). Compared to the single-cell-based states identified by ScregSeg-fi (Figure 1), the ScregSeg-pi (program identification) segmentation frequently led to a subdivision of ScregSeg-fi states, suggesting a refined classification of genomic regions for accessibility patterns across cell types (Figure S2D). ScregSeg-pi genome-wide segmentation is the main resource of our study (Table S6).

To describe the ScregSeg-pi segmentation, we focus on states that show strong association with cell clusters (Figures 2B and S2A). The model identified multiple states that each show clear association with a single cell type (e.g., 29, 4, 14, 24, 10, 23, 21, and 20; Figure 2B). State enrichments around ZFIN and scRNA-seq marker gene sets (Tables S3 and S4) are consistent with those seen for the individual cell-type clusters from our differential analysis (Tables S1 and S2). However, other states capture CREs that are accessible in multiple cell types (e.g., 5, 26, 25, 13, and 1; Figure 2B). For example, ScregSeg-pi states 5 and 18 have a high association with all of the neuronal clusters (clusters 2, 7, 9, and 12). One of the regions with the highest probability of association with these states is around the ascl1b locus (Figure 2B), a key neuronal lineage-determining TF. As this locus is accessible in several clusters, it was not detected by differential accessibility analysis, confirming that ScregSeg-pi is able to capture both broadly acting and highly specialized CREs.

We find evidence of multiple distinct regulatory programs acting in a single cell type. For example, cell-type cluster 14 (Figure 1) is enriched for markers of multipotent caudal precursors with spinal cord, somite, and vascular differentiation potential during body axis extension^45^, ^46^, ^47^, ^48^ (Figure S2E). This cluster strongly associates with three ScregSeg-pi states (25, 26, and 13) whose regions likely constitute distinct regulatory programs behind the known endothelial, neuronal, and myogenic trajectories for caudal precursors, respectively, as evidenced by their shared accessibility with these other cell types (clusters 1/11, 9, and 3/16, respectively) and state marker gene annotations (Figure 2C; Table S3. Enrichment scores for ZFIN-derived annotations per ScregSeg-pi state, related to Figure 2, Table S4. Enrichment scores for scRNA-seq-derived marker genes per ScregSeg-pi state, related to Figure 2, Table S5. Log fold change (logFC) enrichment of genes per ScregSeg-pi state, related to Figure 2). In another example among the neuronal cell types, cluster 12 (midbrain) shows strong associations with 2 states (state 5 and 24). ScregSeg-pi state 5 regions are accessible in all of the neuronal clusters except cluster 6 (differentiating neurons), and are enriched around markers of the neurogenesis cascade, while the cluster 12-specific state 24 includes genomic regions strongly associated with brain spatial identity^49^^,^^50^ (Figure 2C; Table S3. Enrichment scores for ZFIN-derived annotations per ScregSeg-pi state, related to Figure 2, Table S4. Enrichment scores for scRNA-seq-derived marker genes per ScregSeg-pi state, related to Figure 2, Table S5. Log fold change (logFC) enrichment of genes per ScregSeg-pi state, related to Figure 2). This example suggests that it is possible to separate regulatory programs driving differentiation of a specific cell lineage (neurogenesis) from the spatial segregation of brain regions.

**Figure 3:**
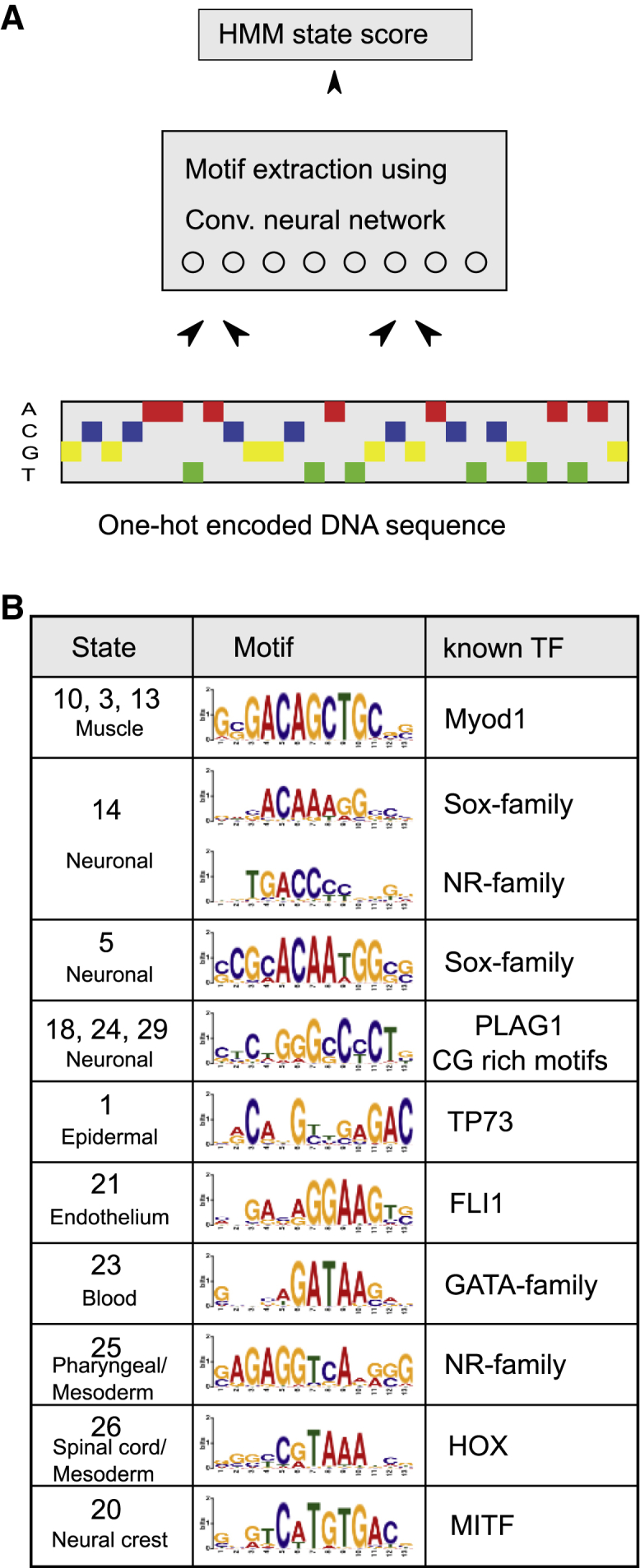
Motif extraction via deep learning (A) A convolutional neural network was used to extract sequence motifs that are predictive of the expected state-read depth score, a combination of the segmentation model’s state calls and the read depth across cells (see STAR Methods). (B) Extracted motifs agree with known motifs of transcription factors implicated in regulating distinct cell-type-specific processes.

Single-cell accessibility measurements have the potential to shed light on the sequence code of transcriptional regulation. Motivated by the success of deep learning approaches for modeling chromatin accessibility and extracting TF-binding events,^11^^,^^51^, ^52^, ^53^ we used convolutional neural networks to predict individual states of the ScregSeg-pi segmentation from the underlying DNA sequence- and extract-associated sequence motifs (Figure 3A; see STAR Methods). We find numerous cases in which the extracted sequence patterns closely resemble motifs of TFs that are known to be active in the cell type associated with the state (Figures 3B and S3). These include a MyoD motif detected in several states enriched for muscle-specific signal, an ETS motif highly similar to that for FLI1 for the endothelial state 21, a GATA motif from the blood-enriched state 23, and in the neural crest state 20, a motif that is likely associated with the microphthalmia-associated TF (MITF). These results orthogonally validate our cell-type annotations and illustrate the potential for ScregSeg-pi segmentation labels to assist future, more detailed models of regulatory sequence code.

### Bulk assays for chromatin architectures reflect single-cell accessibility dynamics

**Figure 4:**
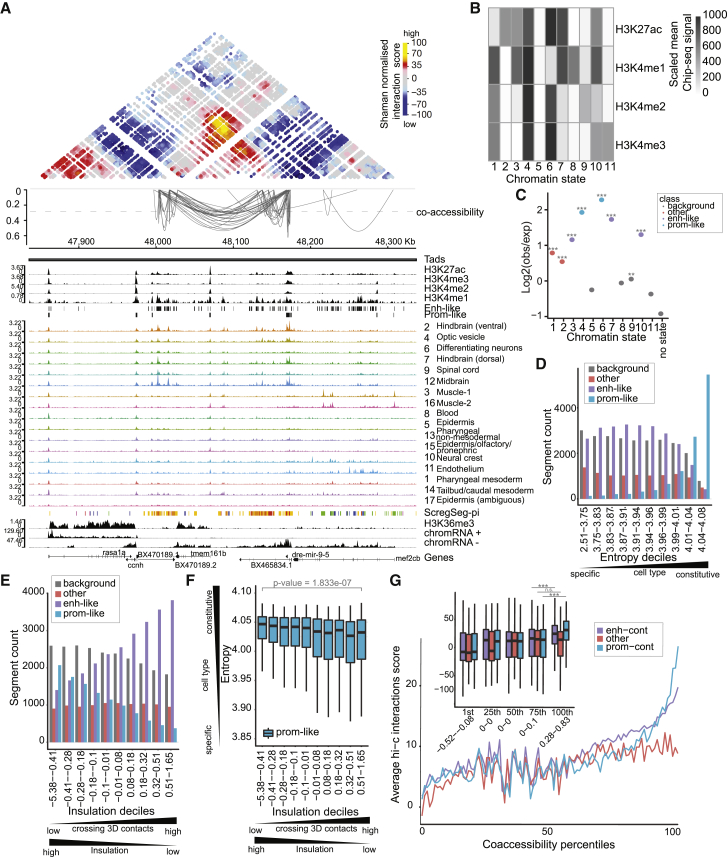
Accessibility dynamics are reflected in histone PTM states and 3D genome organization (A) Browser shot around the dre-mir-9-5 locus showing strong concordance between (from top to bottom) SHAMAN 3D interaction score heatmap, Cicero co-accessibility arcs for scores >0.28 (top 1% score cutoff; dashed line), histone PTM signals and promoter-like/enhancer-like HMM state calls, cluster-collapsed sci-ATAC-seq signals, sci-ATAC-seq segmentation calls, H3K36me3 signal, and nascent chromatin-associated RNA signal. Co-accessibility arcs are clearly enriched between strong interaction regions (orange/yellow in Hi-C heatmap), and these anchor points are clearly marked with enhancer-like and promoter-like PTMs, as captured by the histone PTM states. Co-accessibility is also observed in the sci-ATAC-seq signal tracks and reflected in the similar coloring of the sci-ATAC-seq segmentation calls. (B) A heatmap representing histone PTM chromatin states learned. Each state is a multivariate Gaussian distribution and is plotted as the mean scaled ChIP-seq signal for each PTM. (C) 1-kb segments from the sci-ATAC-seq foreground are classified for their most representative histone PTM state (see STAR Methods), and plotted is the log2 ratio of class occurrence therein compared to class occurrence in all genomic 1-kb bins. The color scale represents the type of histone PTM state as determined from genome-wide frequency and positional enrichment around annotated-TSS proximal and distal segments (Figures S4B–S4D). Stars represent significance from hypergeometric tests for enrichment (for states 1–12, p values are <2.2 × 10^−16^, <2.2 × 10^−16^, <2.2 × 10^−16^, <2.2 × 10^−16^, 1, <2.2 × 10^−16^, <2.2 × 10^−16^, 0.981, 0.00000000641, <2.2 × 10^−16^, 1, and 1, and n values are 6,223, 12,441, 12,871, 6,873, 6,951, 9,283, 12,875, 10,610, 8,231, 11,249, 8,386, and 46,079). (D) Entropy scores (low = cell specific, high = constitutive) for foreground sci-ATAC-seq regions were split into deciles, and within each decile the number of regions for each type of histone PTM state was counted and plotted. (E) *In situ* Hi-C insulation scores for foreground sci-ATAC-seq regions were split into deciles, and within each decile the number of regions for each type of histone PTM state was counted and plotted. (F) *In situ* Hi-C insulation scores for foreground sci-ATAC-seq regions were split into deciles and then split according to their histone PTM type. The entropy score is plotted for the resulting promoter-like histone PTM regions, and the other 3 histone PTM types can be seen in Figure S4I. p value is the result of a Welch’s unpaired t test between the entropy scores for the 1st and 10th insulation deciles with promoter-like chromatin states (mean 4.028477 versus 4.007515, df = 463.39, n = 2,068 and 374, respectively). (G) SHAMAN Hi-C interaction score means (full plot lines) or distributions (inset boxplots) are plotted for pairs of sci-ATAC-seq foreground regions that are >25-kb apart and within the same TAD. Region pairs are split first by Cicero co-accessibility score percentiles and then by having a promoter-like histone PTM state in one or both of the 2 regions (prom-cont), having no promoter-like histone PTM regions but having 1 or 2 enhancer-like PTM regions (enh-cont), or where neither region is promoter-like or enhancer-like (other). Mean lines for all 100 percentiles are plotted for ease of visualization, and boxplot insets for the 1st, 25th, 50th, 75th, or 100th percentiles are shown to give a better sense of the distributions. Counts for each group can be seen in Figure S4J. Stars represent significance from Welch’s unpaired t tests between the 100th and 75th percentiles (prom-cont: mean 26.62588 versus 9.33010, p < 2.2e−16, df = 2,458.4, n = 1,132 and 1,402; enh-cont: mean 19.87832 versus 10.80246, p < 2.2e−16, df = 7514.1, n = 3,829 and 3,740; other: mean 8.958823 versus 9.253661, p = 0.6012, df = 2201.4, n = 1,156 and 1,079).

While accessibility is a highly useful proxy to determine the location of CREs, it does not provide information regarding their function or activity. To further characterize the regulatory landscape of 24-hpf embryos, we performed bulk assays for 3 complementary chromatin modalities: (1) ChIP-seq for 5 histone PTMs commonly used to define and discriminate promoters, enhancers, and gene bodies (H3K27ac, H3K4me1, H3K4me2, H3K4me3, and H3K36me3), (2) *in situ* Hi-C to detect prominent 3D nuclear organization, and (3) chromatin-associated RNA as a measure of nascent transcription (Figures 4A and S4A). ChIP-seq signal for CRE-associated PTMs served as data to infer the parameters of an HMM for chromatin state annotation^54^ with 11 states (Figure 4B; Table S7). These states (referred to now as hPTM states) were further grouped into “promoter-like,” “enhancer-like,” “other,” and “background” types according to overall occurrence (Figure S4B) and spatial patterns around accessible regions proximal and distal to annotated TSSs (Figures S4C and S4D), which are consistent with previous observations in humans, worms, and flies.^54^^,^^55^ ScregSeg-fi regions (Figure 1) show enrichment for promoter-like and enhancer-like hPTM states (Figure 4C; see STAR Methods), suggesting that current models of CRE histone modifications apply also to zebrafish. ScregSeg-fi regions were classified into one of the four hPTM state types (see STAR Methods), confirmed by proximity to annotated TSSs (Figure S4E). Distributions of nascent chromatin RNA-seq counts for regions, not overlapping gene bodies, confirm the utility of our hPTM state types since promoter-like states show elevated RNA levels compared to background states even when located >5 kb from annotated TSSs (Figure S4F; mean log10 counts 2.46 versus 1.93, p < 2.2 × 10^−16^, Welch’s unpaired t test, df = 797.72, n = 660 and 5,914). As the extent of H3K4 methylation likely reflects transcription initiation rates within the associated CRE,^55^^,^^56^ this observation suggests the presence of highly transcribed coding or non-coding transcripts that have eluded annotation efforts, perhaps due to short cytoplasmic half-lives and therefore only being visible in preparations enriched for nascent RNA such as our chromatin RNA-seq.

Previous studies, based on both bulk and single-cell-resolved accessibility measurements^11^^,^^12^^,^^57^ have observed that promoters show high constitutive accessibility across cell types, whereas enhancers are more dynamic and cell specific. To describe the cell type specificity of ScregSeg-fi regions, we calculated the Shannon entropy^12^ for each region across the 17 identified cell types (Figure 1; see STAR Methods). Regions with promoter-like hPTM states show a significant increase in entropy scores compared to background states (mean 4.02 versus 3.89, respectively, p < 2.2 × 10^−16^, Welch’s unpaired t test, df = 35,428, n = 11,438 and 24,149). This is reflected by their accumulation among the most constitutive regions (Figure 4D), and similar trends were observed when considering region proximity to annotated start sites (Figure S4G).

Next, we integrated hPTM states and sci-ATAC-seq measurements with 3D genome organization in the nucleus as measured by *in situ* Hi-C^58^ (see STAR Methods). Gene promoters frequently occur at the boundaries of so-called topologically associating domains^59^^,^^60^ (TADs). A common method for determining TAD boundaries from Hi-C data is the insulation score, which measures aggregate interactions that traverse a given genome position.^61^^,^^62^ Therefore, we calculated insulation scores genome-wide and summarized them for each accessible region. We observed a significant decrease in insulation scores for regions with promoter-like hPTM states compared to background (mean −0.16 versus −0.025, respectively, p < 2.2 × 10^−16^, Welch’s unpaired t test, df = 24,157, n = 11,020 and 23,201) and a significant increase for enhancer-like regions compared to background (mean 0.126 versus −0.025, respectively, p < 2.2 × 10^−16^, Welch’s unpaired t test, df = 48,081, n = 25,448 and 23,201). This is clearly visualized by the accumulation and depletion of enhancer-like and promoter-like regions, respectively, in highly 3D-interacting regions (Figure 4E), trends also observed considering region proximity to annotated TSSs (Figure S4H). Furthermore, constitutively expressed genes are reported to be enriched in TAD border regions.^59^^,^^63^, ^64^, ^65^ Therefore, we explored the relationship between accessibility-based entropy scores, insulation strength, and hPTM state, and observed a significant trend for more insulated, promoter-like regions, and not enhancer-like regions, to be more constitutively accessible (Figures 4F and S4I).

Co-accessibility of pairs of genomic regions within a certain linear genome distance may be an indication of 3D interactions.^9^ To confirm this trend in our dataset, we visualized co-accessibility versus Hi-C interaction scores (Figures 4A and S4A; see STAR Methods) and observed a positive relationship between the two, which was especially significant at the high extremes for pairs containing regions with promoter- or enhancer-like hPTM states (Figures 4G and S4J). At example loci, we clearly observe that strong co-accessibility scores (arcs) link both high-scoring 3D interactions (heatmap) and regions with high cell-type-specific accessibility (Figures 4A and S4A). Furthermore, these co-accessible/interacting regions are assigned to common or related regulatory programs from our ScregSeg-pi analysis (Figure 2), thus showing high concordance between data types and consistency between analysis strategies.

### Sci-ATAC-seq detects cell composition changes in *cloche/npas4l* mutants and identifies novel *cis*-regulatory elements of *npas4l*

**Figure 5:**
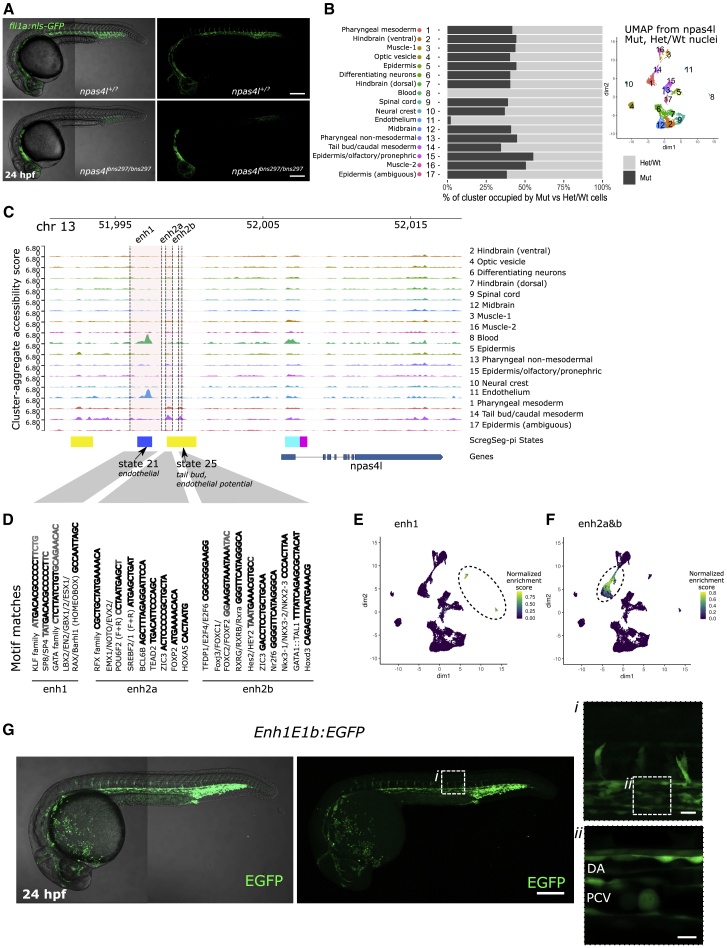
Application of sci-ATAC-seq to *npas4l* embryos reveals unexpected cell-type-specific regulation (A) Representative images of *npas4l* wild-type/heterozygous and homozygous mutants at 24 hpf exhibiting *fli1a:*GFP expression. (B) UMAP representation of the cell-Topic matrix from cisTopic on 8,976 cells, 3,769 homozygous *npas4l* mutants, and 5,207 siblings. Percentages represent the proportion of mutant cells relative to all mutant and sibling cells per density cluster. (C) Summary chromatin accessibility from aggregated cells for each cluster (pseudo-bulks) at the *npas4l* locus. Three cell-type-specific peaks of accessibility are highlighted as putative enhancers enh1, enh2a, and enh2b ∼8–10 kb from the *npas4l* TSS. (D) Motif detection in the enh1, enh2a, enh2b sequences with JASPAR motifs.^69^ Motif scanning at the specific enhancer was with FIMO^70^ and the 20 highest enriched motif sequences are displayed, collapsed per family. Bold black represents a core sequence match shared across the whole family, and gray represents less frequent variations of the motif sequence. (E) Per-cell distribution of accessibility at putative *npas4l* enhancer enh1 (highlighted in C), represented in UMAP space. (F) Per-cell distribution of accessibility at putative *npas4l* enhancer enh2a and enh2b (highlighted in C), represented in UMAP space. (G) *Enh1E1b:*GFP expression at 24 hpf. (i, ii) Single plain optical cross-section through the axial vessels. Scale bars: 200 μm (A and G), 20 μm (i), 10 μm (ii). DA, dorsal aorta, PCV, posterior cardinal vein.

Single-cell maps can characterize the cell dynamics underlying mutant phenotypes and disease.^66^^,^^67^ We reasoned that, conversely, profiling a well-characterized zebrafish mutant phenotype could validate the sensitivity and accuracy of cell type detection from our sci-ATAC-seq data and analysis pipeline. A homozygous mutation in the zebrafish TF gene *npas4l*—historically referred to as *cloche*—results in the development of embryos lacking almost all blood and endothelium, but with elevated cardiomyocyte numbers, while other tissues remain unperturbed.^29^^,^^30^^,^^68^ Harnessing the flexibility to multiplex samples with sci-ATAC-seq, nuclei from homozygous mutant 24 hpf embryos (*npas4l*^*bns297/bns297*^) and their phenotypically wild-type siblings (*npas4l*^*bns297/+*^*, npas4l*^*+/+*^) were tagmented at distinct plate positions, then pooled and processed together for all of the subsequent steps of library preparation (Figure S5A). We then compared the cell composition of the mutant (*npas4l*^*bns297/bns297*^) and phenotypically wild-type sibling (*npas4l*^*bns297/+*^*, npas4l*^*+/+*^) samples, which had been multiplexed and assayed together, by assessing their relative contribution to the clusters derived from all of the batches. As expected, we detected a near-total loss of nuclei from endothelium (cluster 11) and blood (cluster 8) cells in mutants (2.2%, p < 8 × 10^−10^ and 0%, p < 4 × 10^−15^, respectively; versus 42% on average; binomial test; Figure 5B). In contrast, the muscle cluster 16 and epidermal cluster 15 had significantly higher relative contributions from mutant (*npas4l*^*bns297/bns297*^) nuclei (50.65%, p < 0.0002; and 55.41% p < 2 × 10^−6^; versus 42% on average; binomial test), a previously unreported observation. Although the detection of 2 muscle clusters (3 and 16) may have arisen from remaining local batch effects (Figure S1G), a significant increase in muscle cells in the mutant strain is still observed when considering these clusters jointly (48.9%; p < 0.00057; binomial test).

We provide the systematic identification and classification of CREs from our whole-embryo sci-ATAC-seq data as a resource for the targeted exploration of regulation around individual genes. As an example, we observed two distal regions within 30 kb of the *npas4l* TSS annotated as ScregSeg-pi states 21 and 25. These regions were accessible in blood, endothelium, and caudal precursors (cluster 14)—cell types that were depleted or reduced in *npas4l*^*bns297/bns297*^ embryos (Figure 5B)—and lacked long-range interactions with other genomic loci showing equivalent cell-type-specific accessibility (Figures 5C–5F and S5B). We therefore named these putative enhancers of *npas4l* enh1, enh2a, and enh2b. To investigate the putative binding of TFs to these candidate *npas4l* enhancers, we scanned their underlying genomic sequences against the full JASPAR vertebrate database^69^ (Figures 5D and S5C–S5E). We found significant motif matches for blood and mesoderm regulators the KLF family, the GATA family, and LBX2^41^^,^^71^, ^72^, ^73^, ^74^ in the enh1 sequence; RFX family motifs^75^ and ciliogenesis^76^ and mesodermal regulators TEAD2,^77^ NOTO,^78^ EVX2,^79^ and HOXA5 in enh2a; and mesodermally expressed TFs RXRG and FOXC2^80^, ^81^, ^82^ and blood regulators GATA1::TAL1 in enh2b.

Given the clear association between the measured cell-type-specific accessibility for enh1 and the known phenotypes of *npas4l* loss of function, we chose to evaluate its functional activity by cloning 364 bp of the underlying DNA sequence into a reporter construct containing an E1b minimal promoter and EGFP cassette,^83^^,^^84^ generating stable transgenic lines, and examining EGFP expression at 24 hpf. The resulting lines exhibited reproducible EGFP activity in endothelial and blood cells in agreement with the cell-type-specific accessibility of this element and the known phenotypes of *npas4l* loss of function (Figure 5G). As such, we demonstrated the utility of our resource in identifying regulatory elements while also predicting their localized activity. Moreover, sequence analysis can identify candidate upstream transcriptional regulators, which, in the case of *npas4l*, may operate in distinct, cell-type-specific combinations.

## Discussion

We present a multimodal resource for the zebrafish community, which integrates sci-ATAC-seq with bulk histone PTMs and Hi-C data to achieve a genome-wide classification of the regulatory architecture determining transcriptional activity in the 24-hpf embryo. Using our new tool, ScregSeg, we define regulatory programs specific to 1 or more of 17 identified cell types and the prevalent sequences underlying these programs. We find that promoters are mostly constitutively accessible and tend to occur in more insulated 3D neighborhoods and that co-accessible CRE pairs tend to interact in 3D. Sci-ATAC-seq profiling of *npas4l/cloche* mutants validated the sensitivity of our approaches and identified unexpected changes in muscle and epidermal cell populations. Lastly, our ScregSeg-pi classification of multi-cell-type-specific CREs led to the discovery of a novel functional enhancer close to *npas4l* with blood and endothelial specificity. This resource constitutes a solid foundation for future studies in developmental cell biology, systems regulatory genomics, and computational data science, with an immediate direct impact on transgenic reporter gene design, candidate identification for perturbation studies, and regulatory sequence annotation for further developments of predictive models. We encourage the exploration of our data and analyses through the interactive browser (https://scbrowse.mdc-berlin.de/) and UCSC genome browser hub (http://genome.ucsc.edu/cgi-bin/hgTracks?db=danRer11&hubUrl=https://bimsbstatic.mdc-berlin.de/hubs/ohler/scipipe_v4/hub.txt).

In the wake of advances in scATAC-seq experimental methods, a number of analysis strategies have been developed that depend on predefined features and/or focus on various aspects such as cell-type clustering, motif integration, or co-accessibility.^9^^,^^10^^,^^35^^,^^85^^,^^86^ We developed ScregSeg, a novel HMM segmentation approach for analyzing scATAC-seq data, to address (1) the identification of informative features from single-nucleus data (e.g., regions with variable accessibility dynamics) for downstream analysis and (2) the characterization of regulatory programs from cluster-aggregated data, referred to as ScregSeg-fi and ScregSeg-pi, respectively. We show that genomic features derived by ScregSeg-fi facilitate dimensionality reduction, leading to clearly separated cell-type clusters. Benchmarking analysis of ScregSeg-fi suggested that it achieves comparable or sometimes slightly better performance relative to iterative LSI (Figures S1D and S1E; Note S1). ScregSeg-pi analyses identify complex combinatorial accessibility profiles in an unsupervised and unbiased manner (Figure 2). This enabled us to define distinct groups of CREs accessible in cell types that likely act as separable programs, such as the neuronal and mesodermal fates in caudal precursors or the spatial distribution and lineage progression among neuronal clusters. We conducted biological validations, including exploring accessibility profiles at known marker genes, motif analysis within ScregSeg-pi states, integration with other regulatory genomic data types, profiling of a genetic mutant (*npas4l*/*cloche*) with known cell loss phenotype, and transgenic reporter analysis of a small multi-tissue enhancer. These validations position ScregSeg as an important new addition to the toolbox of scATAC-seq analysis methods.

Our integration of single-cell datasets with bulk approaches enabled the identification of global trends and multimodal regulatory principles while addressing the issue of bulk signals being dominated by the most prevalent cell types. Enhancers and promoters share many common characteristics, and the traditional mark distinguishing them, H3K4me3, may simply reflect higher transcription initiation rates.^2^^,^^56^^,^^55^ Our analyses show regions with H3K4me3-containing hPTM states to be often constitutively accessible, suggesting a functional distinction for CREs with this mark. We show that constitutively accessible CREs with promoter-like hPTM states are associated with highly 3D-insulated regions, refining previous observations that TAD borders are associated with constitutively expressed genes during zebrafish development.^65^ Since co-accessible CRE pairs within the same TAD tend to interact in 3D space (Figure 4), our data and analyses suggest the potential of assigning a given zebrafish CRE to its target gene if the promoter is not constitutively accessible, as shown previously in a mammalian system.^9^

The novel *cloche/npas4l* phenotypes highlight the ability of single-cell methods to identify changes in cell numbers during development that may otherwise remain elusive with traditional cell biology methods. The gain in muscle cluster 16 suggests that the lineage commitment of *npas4l* mutant mesodermal precursors is redirected from hematovascular to somite-muscle cell types, resembling a gain of muscle expression from a *npas4l* mutant reporter system (data not shown), and a phenotype observed upon loss of *etsrp*, a direct target gene of *npas4l*.^87^^,^^88^ Accordingly, ScregSeg-pi identified a regulatory program, state 25, that shows accessibility specific to tailbud mesodermal precursors, pharyngeal mesoderm, and endothelial cells (Figure 2B). An increase in an epidermal population is unprecedented and will require further investigation.

We leveraged our highly resolved CRE accessibility profiles to explore the *npas4l* locus, where we observe new putative enhancers—enh1, 2a, and 2b—that exhibit cell-type specificity. The specificity of enh2a/b to caudal precursors supports the importance of *npas4l* in regulating their fate, and we speculate that they may regulate *npas4l* early in the hematovascular fate decision. Meanwhile, the accessibility and validated reporter activity of enh1 in mature blood/endothelial populations, in which its RNA levels are not detectable, supports a purported negative feedback regulation of the transient expression of *npas4l*.^30^ Such a “cloche” enhancer activity has eluded the field for years and will prove a powerful tool to further dissect regulatory networks active in early mesoderm specification. That we were able to detect this enhancer activity in this study highlights the resolution and accuracy of our resource to annotate regulatory activities for follow-up studies.

### Limitations of the study

The focus of this study was to combine single-cell-resolved and multimodal bulk genomics data using advanced computational methods to gain new insights into the zebrafish regulatory genome. Therefore, we limited our samples to a single developmental stage at which many modalities of genomics assays were performed. Increasing the spatial and temporal resolution of such data will expand our understanding of CRE dynamics and function.

Bulk ChIP-seq, Hi-C, and chromatin RNA data were generated from whole embryos to improve *de novo* annotations of the zebrafish genome and identify cross-modality regulatory principles. As similar assays become more commonly performed at single-cell resolution, and from the same single cells, studies will be able to better characterize the relationships between the dynamics of CRE accessibility, histone PTMs, 3D chromatin organization, and nascent RNA production.

The number of cells captured by sci-ATAC-seq was sufficient to identify cell population-specific CREs and to identify changes in cell-type populations associated with the mutant. However, larger sample sizes would be required to reliably resolve cell-type-specific differences in accessibility between the mutant and wild-type samples.

## STAR★Methods

### Key resources table

**Table:** 

REAGENT or RESOURCE	SOURCE	IDENTIFIER
**Antibodies**		

H3K4me1	abcam	Cat#ab8895;RRID:AB_306847
H3K4me2	abcam	Cat#ab32356;RRID:AB_732924
H3K4me3	abcam	Cat#ab8580;RRID:AB_306649
H3K27ac	abcam	Cat#ab4729;RRID:AB_2118291
H3K36me3	abcam	Cat#ab9050;RRID:AB_306966

**Chemicals, peptides, and recombinant proteins**		

Complete Protease Inhibitor	Roche	Cat#11697498001
SUPERase-In RNase Inhibitor	Thermo Fisher Scientific	Cat#AM2696
Trizol	Thermo Fisher Scientific	Cat#15596018
IGEPAL CA-630	Sigma	Cat#I8896
HindIII	NEB	Cat#R3104L
Biotin 14-dCTP	Invitrogen	Cat#19518-018
T4 DNA ligase	Invitrogen	Cat#15224-041
Hind III	NEB	Cat#R0104L
Klenow	NEB	Cat#M0210L
Klenow Exo	NEB	Cat#M0212S
T4 DNA polymerase	NEB	Cat#M0203L
Hercules polymerase	Agilent	Cat#600675
Pronase (Protease from Streptomyces griseus type XIV)	Sigma-Aldrich	Cat#P5147-1G
DSP	Sigma Aldrich	Cat#D3669
Tn5	MDC Protein Production & Characterization Platform, according to Picelli et al., 2014^89^	N/A
DAPI (4’-6-Diamidino-2-Phenylindole Dihydrochloride)	Sigma-Aldrich	Cat#D9542-1MG
EDTA, pH 8.0, ultra pure	Thermo Fisher Scientific	Cat#15575-020
EB buffer	QIAGEN	Cat#19086
BSA	New England Biolabs (NEB)	Cat#B9001S
SDS	N/A	N/A
Triton X-100	N/A	N/A
NEBNext Ultra II Q5 Master Mix	New England Biolabs (NEB)	Cat#M0544L
SYBR Green I	Lonza	Cat#50513
Agencourt AMPure XP beads	Beckman Coulter	Cat#A63881
Phusion HF PCR Mastermix	New England Biolabs (NEB)	Cat#M0531S
BglII	New England Biolabs (NEB)	Cat#R0144S
Gibson Assembly Master Mix	New England Biolabs (NEB)	Cat#E2611L
mMESSAGE mMACHINE	Thermo Fisher	Cat#AM1345
Tricaine	Sigma Aldrich	Cat#304506

**Deposited data**		

Raw data	This paper	GEO: GSE152423
Processed data	This paper	https://bimsbstatic.mdc-berlin.de/hubs/ohler/scipipe_v4/hub.txt)
Processed data	This paper	https://scbrowse.mdc-berlin.de/

**Experimental models: Organisms/strains**		

Zebrafish: AB/TL	Max Delbrück Center Zebrafish facility	N/A
Zebrafish: *Tg(fli1a:nls-GFP)*^*y7*^*npas4l*^*bns297*^	Crossed in Didier Stainier lab from Marass et al., 2019,^88^ Roman et al., 2002, ^90^	N/A
Sea urchin: *S. purpuratus*	David Garfield lab	N/A

**Oligonucleotides**		

Custom transposon oligonucleotides; P7 PCR primers; P5 PCR primers and sequencing primers for sci-ATAC-seq	Cusanovich et al.,^10^ Nature, 2018; Table S12	N/A
Forward primer sequence for cloning Enh1: 5′- AGATGGGCCCTCGAGAGATCTC ACTCTTCAGTCTTCAGTG	Eurofins	N/A
Reverse primer sequence for cloning Enh1: 5′-CCCTCTAGAGTCGAGAGATCTT AATGTGTCCTGCTTCTGC	Eurofins	N/A

**Recombinant DNA**		

E1b-GFP-Tol2	Birnbaum et al., 2012^83^; Li et al., 2009^91^	Addgene plasmid 37845
pT3TS-Tol2	Balciunas et al., 2006^92^	N/A
pTXB1-Tn5	Picelli et al., 2014^89^	Addgene plasmid 60240

**Software and algorithms**		

BEDTools (v2.27.1)	Quinlan and Hall, 2010^93^	http://bedtools.readthedocs.io/en/latest/
MEME Suite (v4.11.3)	Grant et al., 2011^70^	meme-suite.org
samtools (1.9)	Li et al., 2009^91^	N/A
ZenBlue software package	Zeiss	https://www.zeiss.com/microscopy/int/products/microscope-software/zen.html
flexbar (v3.4)	Dodt et al., 2012^94^	N/A
bowtie2 (2.3.4.3)	Langmead and Salzberg, 2012^95^	N/A
ScregSeg	This paper	N/A
cisTopic (0.2.2)	Bravo Gonzalez-Blas et al., 2019^35^	N/A
UMAP (0.2.2.0)	N/A	https://cran.r-project.org/package=umap
densityClust (0.3)	N/A	https://cran.r-project.org/package=densityClust
DESeq2 (1.24.0)	Love et al., 2014^96^	https://bioconductor.org/packages/release/bioc/html/DESeq2.html
scVI (0.5.0)	Lopez et al., 2018^97^	N/A
janggu v0.9.4	Kopp et al., 2020^98^	N/A
keras v2.2.4	Keras, 2015^99^	N/A
TOMTOM (5.0.5)	Bailey et al., 2009^100^	N/A
snakemake (5.2.4)	Mölder et al., 2021^101^	N/A
UMI-tools	N/A	N/A
deepTools (3.1.3)	Ramírez et al., 2016^102^	N/A
JAMM	Ibrahim et al., 2015^103^	N/A
histone PTM ChIP HMM	Duttke et al., 2015^54^; Ibrahim et al., 2018^55^	https://github.com/mahmoudibrahim/hmmForChromatin
FASTX-toolkit	N/A	http://hannonlab.cshl.edu/fastx_toolkit/
Bowtie1	Langmead et al., 2009^104^	N/A
STAR	Dobin et al., 2013^105^	N/A
Cicero	Pliner et al., 2018^9^	https://cole-trapnell-lab.github.io/cicero-release/docs_m3/#installing-cicero
Juicer	Durand et al., 2016^106^	N/A
HiTC	Servant et al., 2012^107^	N/A
Insulation scores	Crane et al., 2015^61^	https://github.com/dekkerlab/cworld-dekker
SHAMAN	Cohen et al., 2017^108^	https://bitbucket.org/tanaylab/shaman/src/default/
ggplot2	Wickham, 2011^109^	N/A
CoolBox	N/A	https://github.com/GangCaoLab/CoolBox

**Other**		

BD FACS Aria III	BD Biosciences	www.bdbiosciences.com
Qubit dsDNA HS Assay	Thermo Fisher Scientific	Cat#Q32854
Bioanalyzer DNA High Sensitivity Kit	Agilent	Cat#5067-4626
NextSeq 500 Sequencing System	Illumina	https://www.illumina.com/
LSM800 observer confocal microscope	Zeiss	https://www.zeiss.com/corporate/int/home.html

### Resource availability

#### Lead contact

Further information and requests for resources and reagents should be directed to and will be fulfilled by the Lead Contact, Scott Allen Lacadie (scott.lacadie@mdc-berlin.de).

#### Materials availability

Plasmids generated in this study are available upon request.

### Experimental model and subject details

All zebrafish maintenance and procedures were conducted in accordance with standard laboratory conditions and animal procedures approved by the local authorities (LAGeSo, Berlin, Germany).

### Method details

#### Embryo preparation for sci-ATAC-seq

For wild-type experiments the AB/TL strain was used. All zebrafish maintenance and procedures were conducted in accordance with standard laboratory conditions and animal procedures approved by the local authorities (LAGeSo, Berlin, Germany). Timed matings were set up between AB/TL adults and embryos were maintained at 28.5°C for 24 hpf from the time of fertilization. Staging and consistency within the clutch was confirmed by morphological criteria.^32^

Chorions were removed by incubating in 15 mL pronase E at 1 mg/ml for 10 min with continuous shaking. Pronase was removed by five washes with 200 mL egg water (60 μg/ml Ocean salt (Red Sea), 3 μM Methylene blue). For the first two wild-type experiments, embryo yolks were removed by placing 100 embryos in 500 μl de-yolking buffer (55mM NaCl, 1.8mM KCl, 1.25mM NaHCO_3_) and pipetting 10 times with a P100 pipette. Embryos were left to sink to the bottom, then de-yolking buffer was removed and 5 washes with egg water were performed. Batches of 25-50 embryos were distributed into 1.5ml eppendorf tubes, egg water removed and snap frozen in liquid nitrogen and maintain at −80°C.

Embryos with mutated *npas4l* alleles were obtained from intercrosses of *npas4l*^*bns297*^ heterozygotes in a *Tg(fli1a:nls-GFP)*^*y7*^ background^88^ maintained under animal protocol B2/1218. Homozygous *npas4l* mutant (*npas4l*^*bns297/bns297*^) embryos were separated from heterozygous and homozygous wild-type siblings (*npas4l*^*bns297/+*^*, npas4l*^*+/+*^) based on the loss of fli1a-GFP+ endothelial cells 24 hours after fertilization. Chorions were removed but yolks were left intact. Embryos were flash frozen in liquid nitrogen, stored at −80°C and transported on dry ice.

#### Nuclei preparation from zebrafish embryos for sci-ATAC-seq

Embryos were thawed in 2 mL of cold lysis buffer^7^ (CLB; 10 mM Tris-HCL, pH 7.5, 10 mM NaCl, 3 mM MgCl_2_, 0.1% IGEPAL CA-630) supplemented with protease inhibitors (Complete Protease Inhibitor Cocktail, EDTA-free, Roche). Embryos were homogenized in a Dounce homogenizer then incubated in cold lysis buffer at 4°C for 1 hour. Nuclei were then strained through 35 micron strainer caps of Corning Falcon test tubes (Thermo Fisher Scientific).

#### Nuclei preparation from sea urchin embryos for sci-ATAC-seq

Sea urchin embryos were S. purpuratus, wild caught in Monterey, California, USA. Embryos obtained 30-48 hpf were fixed for 30 min in 5 mM DSP then quenched with 20 mM Tris pH 7.4 and stored at 4°C. For nuclei preparation, fixed embryos were thawed in 10 mL HB buffer (15 mM Tris, pH 7.4, sucrose 0.34 M, NaCl 15 mM, KCl 60 mM, EDTA 0.2 mM, EDTA 0.2 mM) and then homogenized in a 15 mL Dounce homogenizer 20x with a loose pestle, and 10x with a tight pestle. The homogenate was filtered through Miracloth (Merck Millipore) and rinsed with HB buffer, followed by centrifugation at 3500 g for 5 min at 4°C, discarding the supernatant, twice. The pelleted nuclei were resuspended in cold PBS with 0.1% Triton X-100 and filtered through a 20 μM Nitex membrane, then spun down and resuspended in 1ml CLB.

#### Tn5 transposome preparation for sci-ATAC-seq

Tn5 was generated by the MDC Protein Production & Characterization Platform from Addgene plasmid #60240 according to Picelli et al.^89^ at 1.95 mg/ml with the following minor modifications: buffers lacking Triton X-100 were used for the chitin column and dialysis, and final storage was in 50 mM HEPES-KOH pH 7.2, 0.8 M NaCl, 55% Glycerin, 0.1 mM EDTA, 1 mM DTT. For each experiment 96 uniquely indexed transposon complexes were generated according to Amini et al.^110^ with minor adaptations. First, twenty uniquely indexed transposons were made by annealing a uniquely indexed oligonucleotide (Sigma-Aldrich) containing a Tn5 mosaic end sequence at its 3′ end, to a complementary universal 5′-phosphorylated 19 bp mosaic end oligonucleotide. Oligonucleotides were mixed in a 1:1 molar ratio, giving a final concentration of 100 μm under the thermocycling conditions: 95°C for 5 minutes, cool to 65°C decreasing 0.1°C/second, 65°C for 5 minutes, cool to 4°C decreasing 0.1°C/second. Each annealed oligonucleotide transposon was mixed with Tn5 (1.95 mg/ml) at a ratio of 0.143:1 and incubated for one hour at 25°C. Of the 20 indexed oligonucleotides, 8 contained an adaptor that could be bound by indexed Illumina P5 primers (i5 oligonucleotides) and 12 contained an adaptor that could be bound by indexed Illumina P5 primers (oligonucleotide sequences were obtained from supplementary information of Cusanovich et al.^10^). To make 96 unique transposome complexes each i5 transposome could be mixed with each i7 transposome at a 1:1 ratio in columns 1-12 and rows A-H of a 96 well plate. Transposome complexes were stored at −20°C.

#### sci-ATAC-seq implementation

Our protocol for generating sci-ATAC-seq data was largely following Cusanovich et al.,^10^^,^^11^ with some modifications. Purified nuclei were stained with DAPI (4 μM) and 2500 were sorted into each well of a 96 well plate containing 19 μl of tagmentation buffer (10mM TAPS-NaOH, pH 8.8, 5 mM McCl_2_, 10% DMF, 6.6 mM Tris-HCl, 6.6 mM, 0.066% IGEPAL CA-630) using a BD FACS Aria III (BD Biosciences). For tagmentation, 1 μl of uniquely barcoded Tn5 transposome was added to each well of the 96 well plate containing tagmentation buffer and nuclei. Plates were spun for 30 s at 500 x g and then incubated for 30 minutes at 37°C. Following tagmentation, 40 μl of 40 mM EDTA supplemented with 0.3 mM spermidine was added to each well and the plate incubated for 15 minutes at 37°C. Nuclei and buffer from all wells were pooled in a reagent reservoir and passed through a 35 micron strainer into Corning Falcon test tubes (Thermo Fisher Scientific). DAPI was added and nuclei were sorted again with a BD FACS Aria III. For the second sort, 25 nuclei were sorted into each well of 96-well plates (8-10 plates per experiment) containing 12 μl of nuclear lysis buffer (11 μl of EB buffer (QIAGEN) supplemented with 0.5 μl of 100X BSA and 0.5 μl of 1% SDS). The 96-well plates from the second sort were stored at −20°C until ready for PCR amplification.

Before PCR amplification, each plate was incubated at 55°C for 15 minutes then 1 μl of 12.5% Triton X-100 added per well to quench the SDS. To each well a unique combination of indexed P5 and P7 PCR primers^10^ was added (0.5 μM final concentration each), 10 μl of NEBNext Ultra II Q5 Master Mix (NEB) then immediately amplified in a thermocycler under the conditions: 72°C for 3 minutes, 98°C for 30 s, 18 cycles: 98°C for 10 s, 63°C for 30 s, 72°C for 1 minute, hold at 4°C. Before amplifying a whole plate, the number of cycles was determined from several test wells that were sorted into a separate plate and monitored by qPCR with the addition of SYBR green to the PCR mix. In all experiments here 18 cycles were used.

After PCR amplification, 96-wells of each plate were pooled, cleaned up with DNA Clean & Concentrator-5 columns (Zymo) and then large fragments (above 1000 bp) removed with 1X AMPure beads (Beckman Coulter). The concentration of libraries was measured with Qubit dsDNA HS Assay (Thermo Fisher Scientific) and quality checked with Bioanalyzer DNA High Sensitivity Kit (Agilent).

#### Sequencing of sci-ATAC libraries

Equimolar libraries from each 96 well plate were pools and sequenced with NextSeq500 (Illumina) High Output, 2 × 150 bp loading at a concentration of 1.6 pM. Custom primers^10^ and a custom sequencing recipe^110^ were used to sequence the following read lengths: 110 bp + 45 bp + 110 bp + 39 bp (Read 1 + Index 1 + Read 2 + Index 2).

#### Sci-ATAC-seq analysis

##### Preprocessing

The raw sequences were trimmed using flexbar (v3.4)^94^ with parameters ‘-u 10–min-read-length 50’ using the adaptor sequence ‘CTGTCTCTTATACACATCTG’. Reads were mapped against danRer11 using ‘bowtie2 -X 2000–no-mixed–no-discordant–very-sensitive’. Chromosomes whose names contain the patterns chrM, _random and chrUn were removed from the analysis and only reads with mapping quality of at least 10 were retained. We corrected sequencing errors in barcodes by mapping the sequenced barcodes against the reference barcode universe using bowtie2 with default parameters. Only barcodes with a mapping quality of at least 5 and no more than two mismatches with the reference barcodes were retained. Finally, reads were deduplicated within each barcode using a custom script. A 1:1 mixture of zebrafish and sea urchin nuclei were added to a subset of wells for the first barcoding (tagmentation) step in order to detect barcode collision events.

The barcode collision rate was estimated as described previously using the Birthday paradox.^8^

##### The Scregseg model

Inspired by chromatin segmentation methods,^26^ we developed a hidden Markov model (HMM), called ScregSeg, to segment the genome according to distinct (cross-cell) accessibility profiles. The model takes as input a count matrix representing genome-wide equally sized tiles by either single-cell or cluster-collapsed accessibility counts. Distinct cross-cell accessibility profiles are captured using Dirichlet-Multinomial emission probabilities which represent the states of the model. We utilize the Baum-Welch algorithm to fit the model parameters starting from random initial weights. Multiple restarts are used to avoid poor local minima. After having fit the model, the genome is segmented in the process of state calling using the Viterbi algorithm. The posterior decoding probability per region was computed using the forward-backward algorithm.

##### Defining the regions of interest using ScregSeg-fi

We binned the genome in 1 kb regions and constructed an R x C count matrix where R denotes the number genome-wide 1kb tiles and C denotes the number of cells as follows: Fragments were counted at the midpoint and each entry of the matrix was trimmed to be at most four to mitigate the influence of spurious artifacts. To exclude poor quality cells, we only retained barcodes with at least 1000 and at most 30000 fragments, leading to 21136 barcodes. The resulting count matrix was used as input for Scregseg for the purpose of feature identification (Scregseg-fi).

We utilized an HMM with 50 states and fitted the model for 300 iterations. To avoid poor local optima, we restarted the training process seven times using different random initial weights and eventually used the model that obtains the best overall log-likelihood score.

State calling was performed using the Viterbi algorithm. Only states that each cover at most 1.5% of the genome were retained. These rare states are considered foreground states, while the remaining states were ignored for the downstream analysis (e.g., ambiguous and background states). All regions associated with the foreground states and with posterior decoding probability of at least 0.9 were considered while low confidence regions were eliminated. Finally, we merged bookended regions if they belonged to the same state. This process gave rise to 71,550 regions which were used for the downstream analysis dimensionality reduction step (see below).

To compute the cell-state association heatmap, we determined the fraction of state calls of state i overlapping with accessible regions for a given cell j $a_{ij}=\left(\#\;state\;i\;calls\;overlapping\;accessible\;regions\;in\;cell\;j/\#\;number\;of\;accessible\;regions\;in\;cell\;j\right)$,which represents the observed state frequency associated with cell j. Subsequently, we compute the cell-state association as $log\left(a_{ij}/b_{i}\right)$ where b_i_ denotes the overall state frequency of state i across the genome (Figure 1C). That is, states that occur at a higher frequency in a given cell relative to the overall state frequency are considered to be enriched in the cell.

##### Dimensionality reduction, batch correction and clustering

We constructed a count matrix using the cells across all samples and using the regions of interest defined by ScregSeg-fi (see above). The count matrix was subjected to filtering requiring at least one fragment per region across cells and at least 200 fragments per cell across the ROI regions which led to 23008 barcodes being used for the remaining analysis. We fitted a Latent Dirichlet Allocation model with cisTopic using 30 topics, collapsed Gibbs sampling, a burn-in of 500, and 1000 sampling iterations.^35^

The resulting cell-topic matrix was z score-normalized, and sample specific batch effects were corrected by regressing out the sample-specific information labels using a linear regression model. That is, fitted linear regression models using the ‘lm’ function in R to predict the topic score for each topic t across cells based on the categorical batch label. Afterward, a batch corrected cell-topic matrix is obtained by using the residuals from the model prediction (e.g., the remaining information that cannot be explained by the batch label).

The cell-topic matrix was further used to compute a 2D UMAP embedding. We performed density clustering on the UMAP to group together cells in distinct subpopulations.

We created pseudo-bulk signal tracks based on cells within each density cluster.

Given some query regions (e.g., known marker genes), cell-specific enrichment scores were determined using the AUCell score provided by cisTopic.^35^ These enrichment scores were used to highlight marker accessibility in the UMAP.

##### Differential peak calling

Cluster-specific marker regions were determined by performing one-versus-all differential accessibility analyses using DESeq2^96^ for each density cluster in turn using the regions of interest identified by ScregSeg-fi.

For each cluster, regions with a minimum log2-fold change of one and an adjusted p value of at most 10% were reported as cluster-specific regions. In addition, the top 500 regions with respect to the log-fold change were reported regardless of the above constraints to alleviate the effects of insufficient statistical power for calling regions associated with small cell clusters.

##### Extracting ZFIN-derived annotation features

We compiled body-part specific gene sets using annotation from the ZFIN database.^38^ To this end, we downloaded the gene-body-part association and extracted body-parts present in the 24 hpf developmental stage. We only used annotation data from the publication ids ZDB-PUB-040907-1, ZDB-PUB-051025-1 or ZDB-PUB-010810-1 to ensure consistent quality and remove body parts with less than 6 genes.

##### Extracting scRNA-seq-derived marker genes

We compiled gene sets based on cluster-specific genes for published single-cell RNA-seq data.^25^ To this end, we downloaded the single-cell RNA-seq count matrix along with the cell clustering information from Wagner et al.^25^ We employed scVI^97^ to determine one-versus-all differential gene expression for each cluster in turn based on the 24 hpf single-cell data. We use the top 20 most differential genes per cluster to constitute the scRNA-seq-cluster gene set.

##### Gene enrichment analysis per cluster

For each density-cluster, we determined whether the differentially accessible regions are significantly enriched around the gene sets defined by ZFIN and scRNA-seq data using a hypergeometric test. To this end, we mapped each differentially accessible region to the nearest TSSgene and ensured that each gene was counted only once, in case of multiple marker regions mapping to the same gene. Then we employed a hypergeometric enrichment test to assess whether the differential regions are associated with the gene sets using $\left(\left(\begin{array}{l} n\\ k \end{array}\right)\left(M-n/N-k\right)/\left(M/N\right)\right)$ where k denotes the number of DA regions associated with the gene set, n denotes the geneset size (number of genes in the set), N denotes the number of DA regions associated with any gene and M denotes the total number of genes.

##### Genome segmentation for identifying regulatory programs - ScregSeg-pi

To define input signal tracks, we binned the genome in 500 bp regions. Then we computed a count matrix containing pseudo-bulk (aggregated) fragment counts across the genome with size (number of 500bp regions) x (number of density clusters).

We applied the ScregSeg segmentation model with Dirichlet-Multinomial emission probabilities for segmenting the genome similarly as described above.

We fitted a model with 30 states for 100 iterations using the Baum-Welch algorithm. As above, we repeated model fitting seven times with different random initial weights to minimize the chance of obtaining a poor local optimum. Finally the model with the best log-likelihood score was selected.

We visualize the state-cluster association by normalizing the parameters of the states (that define the emission probabilities) by the total read coverage of the clusters (as large clusters are expected to be covered by more reads than small clusters). Specifically, the parameters for the emission probabilities (expected sufficient statistics) reflect the number of reads in cluster c that are associated with regions of state s, which we normalize by the total number of reads in state s to define the state-specific coverage profile $P_{cs}=\left(number\;of\;reads\;in\;c\;and\;s/number\;of\;reads\;in\;s\right)$. The background coverage describes the number of reads per cluster relative to the total number of reads (regardless of the state assignment), $P_{b}=\left(number\;of\;reads\;in\;c/total\;number\;of\;reads\right)$. We define the state-cell association as $log\left(P_{CS}/P_{b}\right)$.

##### Feature enrichment score per states

In order to assign biological function to states, we developed a statistical test based on the abundance of state calls around the TSS of genes in the gene sets. To this end, we counted the number of state calls $o_{i}$ for each state i across the region defined by the gene set.

The expected number of state counts in a region of the same size N was computed $e_{i}=Np_{i\;}$ where $p_{i}$ denotes the stationary probability of state i of the segmentation model.

We used the enrichment score $\left({\left(o_{i}-e_{i}\right)}^{2}/e_{i}^{2}\right)$ if $\left(o_{i}-e_{i}\right)>0$. Otherwise, the score is zero.

The feature enrichment scores were used to associate functional gene sets with states and for visualization purposes. As gene sets we use the ZFIN and scRNA-seq extracted marker genes as described above and we expanded the regions around the TSSs by ± 10k.

##### Query marker genes for a given state

In order to extract marker genes, we utilized the log-ratio between the proportion of observed state calls covering the gene body ± 10 kb and the proportion of state calls expected by chance in a region of the same size based on the stationary distribution of the HMM. A high positive score indicates an excess of state calls for a particular state relative to its genome-wide state abundance.

##### Testing cell count differences between npas4l mutants and siblings

This test was performed on sci-ATAC-seq profiles from *npas4l* mutant embryos (*npas4l*^*bns297/bns297*^) and their siblings (*npas4l*^*bns297/+*^*, npas4l*^*+/+*^). *npas4l* mutant cells were separated from siblings based on their tagmentation barcodes. We tested for enrichment or depletion of cells from *npas4l* mutants versus siblings using a binomial test for each density cluster. The success probability was determined by (total number of *npas4l* mutant cells) / (total number of *npas4l* mutant and sibling cells).

##### Motif discovery using neural networks

We utilized convolutional neural networks to predict the state probability from the segmentation model weighted by the read counts from the underlying DNA sequence and thereby extracting associated motifs. To this end, we introduce the target score for the regression task as $s_{ij}=d_{ij}\times r_{i}$ for region i and state j where $d_{ij}$ denotes the posterior decoding probability of region i and state j and $r_{i}$ denotes the aggregated read counts for region i. That is, the score captures the cross-cluster accessibility pattern associated with a state while also emphasizing regions with high read counts. For each state, we extracted the top 100k regions according to that score of which we kept the top 15k and the bottom 15k sequences for model training and evaluation. These can be considered as positive and negative sets.

The neural network was implemented using keras v2.2.4^99^ and janggu v0.9.4.^98^ As input to the convolutional neural network we extracted the 500 bp DNA sequences associated with the training and evaluation regions extended by ± 250 bp and converted them to one hot encoding. The network uses a convolutional layer with 100 kernels, 13 bp kernel length and sigmoid activation to scan both strands of the DNA sequence. Subsequently, the maximum activation across the strands is propagated forward and subjected to global max pooling, dropout with 50% and a linear output node. We choose the sigmoid activation in the initial layer due to its relationship with representing Bernoulli random variables, which, after normalization, allows us to approximately interpret the kernel weights as log-likelihood ratios and thus position weight matrices.

The network was trained on all regions, except for regions on chromosome 1 and 5 which were used as a validation set. Training was performed using ADAM by minimizing the mean absolute error for at most 300 epochs with batch size 32 and early stopping with a patience of 20 iterations. After model fitting, the 10 kernels whose maximum hidden activations per sequence after the first convolution layer individually correlated most with the state-specific score were extracted, normalized to represent PWMs and reported as *de novo* motifs.

These motifs were matched against motifs from JASPAR 2018,^69^ non-redundant vertebrates using TOMTOM.^100^

#### ChIP-seq

Embryos were collected 24 hpf, dechorionated (as described in embryo preparation for sci-ATAC-seq), briefly washed in PBT (0.1% Triton X-100 in PBS) and fixed in 0.5% formaldehyde (Carl Roth #4979.1) in PBS for 15 minutes as previously published for *D. melanogaster*^111^ with minor modifications: heptane was not added to the fixation buffer since unlike *D. melanogaster* zebrafish does not have a cuticula. They were washed in PBT-Glycine (PBS, 125mM glycine, 0.1% Triton X-100) and twice in PBT (PBS, 0.1% Triton X-100) and snap frozen. Nuclei were extracted according to^112^: embryos were resuspended in 2 mL cell lysis buffer (10mM Tris–HCl pH 7.5, 10mM NaCl, 0.5% IGEPAL (CA-630, Sigma, I8896), homogenized on ice for 15 min (dounced 20 times with a loose pestle and 10 times with a tight pestle), spun at 2000 g for 5 min at 4C. Nuclei were then lysed for 10 min on ice in nuclei lysis buffer (50mM Tris–HCl pH 7.5, 10mM EDTA, 1% SDS, protease inhibitor cocktail), two volumes of IP dilution buffer (16.7mM Tris–HCl pH 7.5, 167mM NaCl, 1.2mM EDTA, 0.01% SDS, protease inhibitor cocktail) were added and aliquots were sonicated for 16 cycles (30 s ON, 30 s OFF, on high setting) in a Bioruptor Plus (Diagenode) to achieve a DNA fragment size below 500 bp. ChIP was performed using True MicroChIP Kit (Diagenode #C01010130) according to the manufacturer’s instructions with the following modifications: primary antibody was incubated at 4°C overnight and the reverse crosslinking was done overnight. The following antibodies were used: H3K4me1 (abcam #ab8895), H3K4me2 (abcam #ab32356), H3K4me3 (abcam #8580), H3K27ac (abcam #ab4729) and H3K36me3 (abcam #ab9050). The library was prepared using NEXTflex qRNA-Seq Kit v2 (BioScientific #5130-12, discontinued) according to the instructions for qChIP-Seq and paired-end sequencing (2 × 75nt) was performed on a NextSeq 500/550 using a HighOutput v2 Kit for 150 cycles (Illumina #FC-404-2002, discontinued).

#### ChIP-seq processing

Processing steps were implemented within the Snakemake framework.^101^ UMIs were extracted from paired-end reads using UMI-tools^113^ and mapped to the danRer11 genome assembly using Bowtie2;^95^ -X 2000–no-discordant–no-mixed). Mapped reads were sorted, indexed, and converted to .bam format with samtools^91^ then filtered for MAPQ 30 and deduplicated using UMI-tools. Input-subtracted .bigwig for visualization (–operation subtract–binSize 50–scaleFactorsMethod None–normalizeUsing CPM–smoothLength 250–extendReads) and .bedgraph for HMM (–operation subtract–binSize 1–scaleFactorsMethod None–normalizeUsing CPM–extendReads; see below) tracks were generated using deepTools.^102^ Reads were converted to bedpe files using bedtools.^93^ Peaks were called using JAMM^103^ considering both replicates separately (-r window -e 1 -b 250 -t paired).

#### Histone PTM HMM

Signals and peak calls from histone PTM ChIP-seq data were used as input for generating a HMM segmentation model as previously described.^54^^,^^55^ Briefly, using bedtools intersect and map,^93^ genome-wide 10 bp resolution tracks were generated for each factor such that places where peaks were called were assigned values from ChIP-seq signal files and where no peaks were called assigned values of zero. These signal tracks for chromosomes 1 and 2 were then used as input to bw.r (https://github.com/mahmoudibrahim/hmmForChromatin) to learn the model and the resulting model used to decode the rest of the genome states using decoding.r (https://github.com/mahmoudibrahim/hmmForChromatin). The model was learned with increasing number of states until patterns of state coverage around segments proximal to annotated TSSs resembled previously observed patterns across metazoans,^55^ leading to the selection of a model with 11 states plus one background state where no ChIP-seq peaks were called and/or ChIP-seq signals were £ 0.

To classify sci-ATAC-seq regions as being enriched for a given histone PTM state, we developed a score where the state coverage for a given region (obtained using bedtools annotate) is divided by the sum of the observed state coverage for the region set, and then took the log of the ratio between this normalized coverage and the expected coverage of the state for that region size given the genome-wide state probabilities. The region was classified as the state with the highest score. For Figure 4C, we applied this classification to all genome-wide 1 kb bins, counted the number of classifications for each state, and divided that number by the total number of 1 kb bins to get expected state classification fractions. We then applied the classification to the unmerged sci-ATAC-seq foreground 1 kb bins, calculated the observed classification fractions for each state, and plotted the log2 ratio between this number and the expected state classification fractions.

#### Cellular fractionation

Embryos were collected 24 hpf, dechorionated (as described in embryo preparation for sci-ATAC-seq) and homogenized on ice in buffer N (10 mM HEPES pH 7.5, 250 mM sucrose, 50 mM NaCl, 5 mM MgCl_2_, 1 mM DTT, 1X Complete Protease Inhibitor (Roche #11697498001) and 20 U/ml SUPERase-In RNase Inhibitor (Thermo Fisher Scientific #AM2696)) using a Dounce homogenizer. After allowing the debris to settle for 5 minutes on ice the supernatant was then washed in PBS, loaded on a sucrose cushion (10 mM Tris pH 7.4, 150 mM NaCl, 24% sucrose), centrifuged at 1000 g at 4°C and further fractionated according to Conrad and Ørom,^114^ nuclei were briefly washed in PBS-EDTA (PBS, 0.5 mM EDTA) resuspended in 250 ul of glycerol buffer (20 mM Tris pH 7.4, 75 mM NaCl, 0.5 mM EDTA, 50% Glycerol, 20 U/ml SUPERase-In RNase Inhibitor) and 250ul of Urea buffer (10 mM Tris pH 7.4, 1 M Urea, 0.3 M NaCl, 7.5 mM MgCl_2_, 0.2 mM EDTA, 1% Igepal CA-630, 20 U/ml SUPERase-In RNase Inhibitor) was immediately added, vortexed and incubated on ice for 2min and spun at 13000 g for 2min. Chromatin pellet was briefly washed in PBS-EDTA (PBS, 0.5 mM EDTA) and resuspended in Trizol using a 21 gauge needle and syringe. Total and chromatin RNA was extracted using Trizol (Thermo Fisher Scientific #15596018) and Direct-zol RNA MiniPrep Kit (Zymo Research #R2052) according to the manufacturer’s instructions. The library was prepared using NEXTflex Rapid Directional qRNA-Seq Kit (BioScientific #NOVA-5130-01D) according to the manufacturer’s instructions and paired-end sequencing (2 × 75 bp) was performed on a NextSeq 500/550 using a HighOutput v2 Kit for 150 cycles (Illumina #FC-404-2002, discontinued).

#### Chromatin RNA-seq processing and analysis

Unique molecular identifiers (UMIs) were extracted from .fastq files using UMI-tools^113^ and reads trimmed using fastx_trimmer from the FASTX-toolkit (http://hannonlab.cshl.edu/fastx_toolkit/). Reads were then filtered for ERCC spike-in reads and rRNA by mapping to a custom index with Bowtie 1.^104^ Trimmed, filtered, reads were then mapped using STAR.^105^ Mapped .bam files were then subjected to PCR deduplication using UMI-tools,^113^ followed by conversion to .fastq and remapping with STAR to generate final mapped files and normalized coverage tracks. For sci-ATAC-seq region chromatin RNA quantification, coverage tracks were created for each genome strand using deepTools^102^ and then summed within a 5 kb window centered around the segment midpoint using bedtools map.^93^

#### sci-ATAC-seq entropy

Foreground sci-ATAC-seq regions were counted for reads from density cluster-collapsed .bam files using bedtools multicov. A pseudocount of 1 was added to the matrix before per-cluster depth normalization. Then the Shannon entropy was calculated for each region’s normalized count vector across the clusters using the following equation:

$$SE\;=\;-\overset{n}{\underset{i}{\sum }}p_{i}log_{2}p_{i}$$

Where p is the probability of ATAC-seq signal in cluster i for a given region and n is all the sci-ATAC-seq clusters.

#### Co-accessibility

Foreground sci-ATAC-seq regions were measured for co-accessibility using Cicero^9^ with cisTopic topic probabilities and topic-based UMAP coordinates (see above Method details “Dimensionality reduction, batch correction and clustering”) as reduced dimension information, but otherwise with default parameters.

#### *In situ* Hi-C

Embryos were collected 24 hpf, dechorionated, fixed in 1% formaldehyde in PBS, quenched and washed as in Bonn et al.^111^ Nuclei were extracted according to Bogdanović et al.^112^ using the cell lysis buffer (10 mM Tris–HCl pH 7.5, 10 mM NaCl, 0.5% IGEPAL (CA-630, Sigma, I8896) and a Dounce homogenizer as described in the Chip section.

Hi-C library preparation was performed as previously described^58^ with modifications. In brief, 25 × 10^6^ isolated nuclei were divided in 5 aliquots and digested overnight with 1500U of HindIII (NEB, #R3104L) per aliquot. After biotin-fill in, proximity ligation was carried out in each aliquot with 100 units of T4 DNA ligase (Invitrogen) at 16°C overnight. DNA was purified by reverse crosslinking and DNA precipitation, and biotinylated nucleotides were removed from unligated fragments ends with 1U T4 DNA polymerase per μg DNA for 4 hours at 12°C. DNA was sonicated to 300 – 500 bp, using the Bioruptor Plus and size selected for fragments between 300 and 500 bp with AMPure XP beads (Beckman Coulter). Biotinylated DNA fragments were pulled down with MyOne Streptavidin C1 beads (Invitrogen), end-repaired, A-tailed, and TruSeq sequencing adapters were ligated to the DNA fragments with 15U T4 DNA ligase (Invitrogen) overnight at 16°C, shaking at 750 rpm. Adaptor-ligated DNA was amplified for 6-8 cycles using Herc II Fusion DNA polymerase (Agilent). PCR products were purified with AMPure XP beads and subjected to Illumina paired-end sequencing (2 × 75 bp).

#### *In situ* Hi-C analysis

Processing steps were implemented within the Snakemake framework.^101^ *In situ* Hi-C reads were initially processed using the Juicer pipeline.^106^ TADs were called using the insulation method^61^ with default settings (–is500000–nt0–ids250000–ss0–immean) after dumping valid interactions at 25 kb binned resolution using juicer-tools and converting formats using HiTC.^107^ For scoring Hi-C interactions, valid interactions were dumped by juicer-tools at fragment resolution, filtered to remove interactions less than 20 kb apart and format converted using custom scripts, and then subjected to shuffling and scoring using the SHAMAN method.^108^

Insulation scores for sci-ATAC-seq regions were calculated as the mean signal from 40 kb windows surrounding the midpoints of the segments using bedtools slop and map.^93^

For Hi-C/co-accessibility analysis, foreground sci-ATAC-seq regions used for clustering were measured for co-accessibility (see above). Region pairs were then filtered to remove pairs less than 25 kb (custom scripts) apart and to be within the same TADs using bedtools pairtobed.^93^ Filtered region pairs were then assigned a SHAMAN interaction score,^108^ using a slightly modified version of “shaman_generate_feature_grid” (https://bitbucket.org/tanaylab/shaman/src/default/) in which pair relationships are pre-determined and not re-calculated. For all filtered region pairs, the maximum SHAMAN score was retrieved within a 5 kb grid around the interaction point from the region midpoints.

#### Data visualization

All plots for Figures 4 and S3 were generated using ggplot2,^109^ except for the Hi-C heatmaps which were plotted using “shaman_plot_map_score_with_annotations” (https://bitbucket.org/tanaylab/shaman/src/default/), the browser shots which were plotted using CoolBox (https://github.com/GangCaoLab/CoolBox), the co-accessibility arcs which were plotted using the built-in Cicero plotting function,^9^ the histone PTM state coverage around annotated regions which was plotted from built-in R plot function, and the histone PTM state/mark enrichment heatmap which was plotted by default from the HMM scripts (https://github.com/mahmoudibrahim/hmmForChromatin).

#### Motif scanning in putative enhancers of npas4l

Sequences form putative enhances were obtained from their genomic coordinates using bedtools getfasta v2.27.1^93^ with reference genome danRer11 described above. A 0-order Markov local background was generated for each putative enhancer from its sequence plus its 250 bp flanking sequences using fasta-get-markov from the MEME Suite 4.11.3.^100^ Each putative enhancer sequence was then scanned for matches to motifs from the JASPAR vertebrate database^69^ using FIMO from the MEME Suite 4.11.3^70^ with the aforementioned background.

#### Enhancer cloning and transgenesis

Npas4l enhancer candidate “enh1” was PCR amplified with Phusion HF PCR Mastermix (NEB #M0531S) from genomic DNA using primer sequences 5′- AGATGGGCCCTCGAGAGATCTCACTCTTCAGTCTTCAGTG and 5′-CCCTCTAGAGTCGAGAGATCTTAATGTGTCCTGCTTCTGC. The product was cloned into E1b-GFP-Tol2^83^ (Addgene #37845) after digesting the plasmid with BglII (NEB, #R0144S) using Gibson Assembly Master Mix (NEB, #E2611L). The insert was confirmed with Sanger sequencing to be TCACTCTTCAGTCTTCAGTGTCTGATCTCTGGTCCGGGTCTGATCATCTGTAATGCTGCTTGTGACTCCTCAGCCAATCAGCAGAAGGGGGCGTGTCATAACTGTCGTGGGAATATGACAGGCGTTATGAAGCGTTATGAGCTCTGTAGAGGAGCAGTGCTGACTACAGCCTGACCACCAGCACTGCAGCGCACGCGAGTGTGTGTGTGTGTGTGTGTGTGTCTAGTGTGTGTGTTCTGCACAGATAAGAGCTCTACAGGAAGTCATCACACATGAAGATTTCCTGAACACAGCACTCATGCAGGAGCAGGAAGAGGAAACACACACAAACACACACATTACTGGCAGAAGCAGGACACATTAA. mRNA of the *Tol2* transposase was *in vitro* transcribed from XbaI-linearized pT3TS-Tol2^92^ using the mMessage mMachine kit (Ambion). 25 pg enh1-e1b-GFP plasmid DNA alongside 25 pg *Tol2* mRNA were injected into zebrafish zygotes and the injected embryos screened for fluorescence at 24 hpf.

#### Fluorescent embryo imaging

Embryos were embedded laterally in 1% low-melting agarose solved in eggwater. Living embryos were anaesthetized with 0.01% tricaine prior to embedding and stayed under anesthesia during microscopy. The images were acquired using a LSM800 observer confocal microscope (ZEISS) and processed using the ZenBlue software package. Only linear adjustments were used.

Brightfield-like images were generated using ESID combined with enhanced depth of focus. The channel was then added to the orthogonal projection of the fluorescent channels. For whole embryo imaging, tile scans of z stacks with a Pln Apo 10x/0.45 DICII objective (ZEISS) were stitched with the ESID as reference channel.

Images of the trunk region were acquired using the C Apo 40x/1.1 W DICIII (ZEISS). For optical sectioning through the axial vessels, an Airyscan detector, followed by 2D airyscan processing was used. As anatomical landmark, we kept the yolk extension in the field of view.

### Quantification and statistical analysis

Details on statistical tests can be found in the Results, figure legends, and Method details sections.

### Additional resources

The manuscript is accompanied by an interactive web-browser for single-cell ATAC-seq data which is available at https://scbrowse.mdc-berlin.de. In addition, we provide a track hub at (http://genome.ucsc.edu/cgi-bin/hgTracks?db=danRer11&hubUrl=https://bimsbstatic.mdc-berlin.de/hubs/ohler/scipipe_v4/hub.txt). The source code for Scregseg is available at https://github.com/BIMSBbioinfo/scregseg.

## Data Availability

The manuscript is accompanied by an interactive web-browser for single-cell ATAC-seq data at https://scbrowse.mdc-berlin.de. In addition, we provide a track hub at http://genome.ucsc.edu/cgi-bin/hgTracks?db=danRer11&hubUrl=https://bimsbstatic.mdc-berlin.de/hubs/ohler/scipipe_v4/hub.txt. All raw data is available on the NCBI Gene Expression Omnibus (GEO) with accession GSE152423. The processed data is available as a UCSC hub at http://genome.ucsc.edu/cgi-bin/hgTracks?db=danRer11&hubUrl=https://bimsbstatic.mdc-berlin.de/hubs/ohler/scipipe_v4/hub.txt. The source code for ScregSeg is available at https://github.com/BIMSBbioinfo/scregseg. Any additional information required to reanalyze the data reported in this paper is available from the lead contact upon request.

## Supplemental information

Document S1. Note S1 and Figures S1–S5

Table S1. Enrichment scores for ZFIN-derived annotations per cluster, related to Figure 1

Table S2. Enrichment scores for scRNA-seq-derived marker genes per cluster, related to Figure 1

Table S3. Enrichment scores for ZFIN-derived annotations per ScregSeg-pi state, related to Figure 2

Table S4. Enrichment scores for scRNA-seq-derived marker genes per ScregSeg-pi state, related to Figure 2

Table S5. Log fold change (logFC) enrichment of genes per ScregSeg-pi state, related to Figure 2

Table S6. Bed file with ScregSeg-pi foreground segmentations and state probabilities, related to Figure 2

Table S7. Bed file with hPTM states, related to Figure 4

Document S2. Article plus supplemental information
