# Supplementary material for: Single-cell-resolved dynamics of chromatin architecture delineate cell and regulatory states in zebrafish embryos
Source: Cell Genom. 2022 Jan 13;2(1):100083. doi: 10.1016/j.xgen.2021.100083 (PMC9903790; doi:10.1016/j.xgen.2021.100083)
Supplement: Document S1. Note S1 and Figures S1–S5 [file mmc1.pdf]

Cell Genomics, Volume *2*

Supplemental information

Single-cell-resolved dynamics of chromatin architecture delineate cell and regulatory states in zebrafish embryos

Alison C. McGarvey, Wolfgang Kopp, Dubravka Vučićević, Kenny Mattonet, Rieke Kempfer, Antje Hirsekorn, Ilija Bilić, Marine Gil, Alexandra Trinks, Anne Margarete Merks, Daniela Panáková, Ana Pombo, Altuna Akalin, Jan Philipp Junker, Didier Y.R. Stainier, David Garfield, Uwe Ohler, and Scott Allen Lacadie

Supplementary Note

Comparing iterative LSI and ScregSeg-fi on zebrafish sci-ATAC-seq dataset

We utilized the ArchR package[1] (v 1.0.0) to visually compare how different feature selection approaches influence the cell-type characterization on zebrafish sci-ATAC-seq data (this dataset). To this end, we followed the ArchR tutorial to load our final set of filtered bam files, remove poor quality cells and likely doublets. We conducted feature selection and dimensionality reduction using the built-in iterative LSI method with default parameters. In addition, we loaded the ScregSeg-fi-derived features (which were also used for the main analysis of this manuscript) followed by dimensionality reduction with the built-in LSI (using all features and without iterative feature refinement). That is, except for the feature selection step, pre- and post-processing were performed in the same way within the ArchR package to ensure comparability. This includes using LSI for dimensionality reduction instead of cisTopic (which was used in the main analysis). Finally, the resulting reduced dimensionalities were subjected to the UMAP algorithm for visual comparison of the methods.

Visual inspection of the UMAP embeddings reveals that the larger cell populations (e.g. neuronal cells) can be detected with iterative LSI as well as ScregSeg-fi features. However, some small cell populations, including cluster 10 (neural crest) or cluster 11 (endothelium) are unravelled with ScregSeg-fi, but seem to be merged into other cell populations when using iterative LSI (Figure S1E). This analysis suggests that the usage of ScregSeg-fi derived features in our main analysis is indeed adequate.

Benchmarking analysis of ScregSeg-fi on publicly available data

We employed a recently published scATAC-benchmarking framework[2] to quantitatively compare the influence of feature selection on cell-type identification on three previously published datasets: Hematopoiesis[3], adult mouse tissue cells[4] and 10X Genomics peripheral blood mononuclear cells. In particular, we used the benchmarking framework 1) to compare the robustness/sensitivity of the cell-type identification results for different informative state and region selection criteria using Scregseg-fi and 2) to compare Scregseg-fi and iterative LSI-derived feature selection.

To ensure compatibility with the benchmarking framework, we prepared the binarized count matrices (with adjustments for the fixed-size genome-wide bins; e.g. using 500bp and 1kb bins) according to the publicly available scripts for running LSI (labelled as Cusanovich2018 in [2] for all datasets[2]. Regions without any fragment counts and regions from sex chromosomes were discarded. We applied iterative LSI as implemented in ArchR on count matrices with 500bp resolution using default settings to obtain low-dimensional representations of the cells. For ScregSeg-fi, we fitted a 50-state HMM using 100 iterations and 1kb resolution count matrices. The HMM was then used to identify a set of informative states and extract their associated regions (with posterior decoding probability of at least 0.9) for dimensionality reduction (see below). To facilitate comparability to iterative LSI, we used the LSI implementation from ArchR with default parameters (but without iteration) to perform dimensionality reduction on the ScregSeg-fi selected features. The low-dimensional feature matrices were subjected to clustering using Louvain clustering, k-means clustering and hierarchical clustering. Finally, the clustering results were scored against ground truth labels (for Buenrostro et al. 2018 and Cusanovich et al. 2018), using the adjusted Rand score (ARI) and the adjusted mutual information (AMI). High ARI and AMI scores indicate that clusters reflect known cell types. For the 10X Genomics dataset, the residual average Gini-index (RAGI) was determined to assess

the separation between known marker genes compared to a set of housekeeping genes (as ground truth cell-types are lacking for this dataset). For each feature matrix, the best score across the three clustering algorithms was reported.

First, we focused on comparing different feature selection criteria using Scregseg-fi (e.g., different numbers of foreground states and regions). We identify putative informative states based on the number of associated state assignments throughout the genome and select those states with the fewest assigned regions. In some cases it may be difficult to decide exactly which states ought to be considered as informative, for instance, because of ambiguous or conflicting state characteristics (as described in the main text; Figure 1C-F, S1C). Therefore, we assessed how different numbers of selected states (e.g., selecting N=5, 10, 15, 20 and 25 out of the 50 states which were assigned to the smallest number of regions across the genome) influence cell-type identification. We find that with increasing numbers of selected informative states, the cell-type identification scores initially improve (Figure S1D). However, after having selected approximately 10-20 states the performance plateaus and adding more states does not further improve the results substantially for all three datasets. This range contains the number of ScregSeg-fi states (N=17) that were used for the main analysis and suggests that even if the exact number of informative states is difficult to discern, largely similar performances for the downstream analysis can be achieved with a range of selections of informative states. This observation highlights the robustness of the ScregSeg-based feature identification approach (Figure S1D).

Next, while it is possible to simply use all regions associated with the informative states for the downstream analysis (as we do in the main analysis of this work), in some applications, due to the inherent similarity of regions associated with a given state, it might be desirable to consider using only a subset of assigned regions per state for the dimensionality reduction step. This way the total number of regions can be limited (e.g., to avoid too large feature sets) and computational requirements for the downstream analysis can be reduced. Accordingly, we

tested the use of different numbers of regions per state (at most 500, 1000, 1500, 2000, 3000, 4000, 5000, 7000 regions per state with the highest coverage across cells) and observe that for the Buenrostro 2018 and Cusanovich 2018 datasets, the cell-type identification performance seems to saturate after having selected about 2000-4000 regions per state. A similar observation can be made for the 10X Genomics datasets, even though the performance appears to be more variable in this case (Figure S1D). This suggests that reasonable cell-type clustering performance can be reached with a subset of representative cell-type specific regions rather than having to use all of the cell-type specific regions.

Finally, in comparison to iterative LSI, we find that ScregSeg-fi seems to achieve similar performances (e.g. on et al. Buenrostro 2018) or slightly better performances (e.g. on Cusanovich et al. 2018 and the 10x Genomics dataset) which underscores the validity of the ScregSeg-fi approach for feature identification.

ScregSeg-fi and ScregSeg-pi correspondence

While ScregSeg's HMM model can be used to characterize the genome based on single-cell resolution, we opted to skip interpreting these states directly and merely use them to define the putative informative feature sets (which we termed ScregSeg-fi). We continued with the informative features by applying a separate dimensionality reduction step using cisTopic. While cisTopic requires a pre-defined feature set, we empirically observe that its low-dimensional feature representation leads to improved clustering and cell-type identification results compared to when the ScregSeg-fi states were interpreted directly (data not shown). Presumably this is due to the fact that it does not have to cope with genomic background noise to the extent ScregSeg-fi needs to.

However, after cell clusters have been identified, we can aggregate the signal tracks with each cell group (cluster) and operate ScregSeg on the cluster-collapsed accessibility tracks

(which we refer to as ScregSeg-pi). In this way one benefits from signal enhancement that is brought about by the cell aggregation and the preceding dimensionality reduction step.
We find evidence for the refinement of the regulatory landscape characterization using ScregSeg-pi compared to ScregSeg-fi by exploring the overlap of the respective state calls. ScregSeg-fi states are frequently subdivided into multiple ScregSeg-pi states (Figure S2D). A notable example is given by ScregSeg-fi state_2 which becomes subdivided into distinct ScregSeg-pi states: state_21, reflecting endothelial cells and state_23 reflecting blood cells. An explanation for this could be that ScregSeg-fi, since it operates on the original high-dimensional single-cell resolution, is more prone to getting stuck in local minimal when identifying its states. This could cause the model to express several distinct cell-types using a single cross-cell accessibility representation (state). However, when using ScregSeg with cluster-collapsed accessibility tracks, the likelihood of obtaining poor local minima is reduced, because of the reduced dimensionality of the problem which in turn frequently leads to a fine-tuning of the regulatory landscape.

Computational requirements of ScregSeg

ScregSeg enables genome segmentation based on single-cell or pseudo-bulk resolution accessibility tracks. The computational requirements differ for these two use cases.

On single-cell resolution tracks (as used for ScregSeg-fi here), ScregSeg operates on sparse matrix format (scipy.sparse) to achieve memory efficiency. The memory footprint depends on the cell numbers, the genome binning resolution and the sparsity of the dataset. For instance, this will lead to a memory requirement of approximately 300 Mb for the hematopoiesis dataset[3] (~2000 cells, 1kb genome-wide binning) and 16 Gb for the mouse tissue dataset[4] (~80000 cells,

1kb genome-wide binning). For large future datasets that do not fit into the memory, it is possible to run the segmentation on a subset of cells.

Regarding the runtime, the main computational bottleneck is given by the parameter fitting procedure (Baum-Welch algorithm), which requires to repeatedly scan the genome to collect the parameter updates and infer the most probable states for each region in the genome. We implemented a parallelized parameter fitting procedure in order to speed up the runtime behaviour. This is achieved by splitting the genome into distinct non-overlapping chunks, each one being processed in parallel to obtain the partial sufficient statistics. Subsequently, the results are aggregated across the chunks. Using the parallelized parameter update procedure, a 50-state HMM can be fitted on ~80000 cells (e.g. the Cusanovich et al. 2018 dataset) using 30 cores in about 3 hours.

The segmentation applied to pseudo-bulk tracks (as used for the scregseg-pi step) differs from the single-cell track segmentation primarily by the lower memory requirements. That is, by taking advantage of the preceding dimensionality reduction step, the number of pseudo-bulk tracks is usually several orders of magnitude smaller than the number of single cells, which results in a much reduced memory footprint of the algorithm. This in turn enables the application of ScregSeg even for very large future datasets, because it’s memory requirements does not depend on the number of single-cell cells but rather on the number of clusters/subpopulations that were identified.

References

1. Granja, J.M., Corces, M.R., Pierce, S.E., Bagdatli, S.T., Choudhry, H., Chang, H.Y., and Greenleaf, W.J. (2021). ArchR is a scalable software package for integrative single-cell chromatin accessibility analysis. Nat. Genet. *53*, 403–411.

2. Chen, H., Lareau, C., Andreani, T., Vinyard, M.E., Garcia, S.P., Clement, K.,

Andrade-Navarro, M.A., Buenrostro, J.D., and Pinello, L. (2019). Assessment of computational methods for the analysis of single-cell ATAC-seq data. Genome Biol. *20*, 241.

3. Buenrostro, J.D., Corces, M.R., Lareau, C.A., Wu, B., Schep, A.N., Aryee, M.J., Majeti, R., Chang, H.Y., and Greenleaf, W.J. (2018). Integrated Single-Cell Analysis Maps the Continuous Regulatory Landscape of Human Hematopoietic Differentiation. Cell *173*, 1535–1548.e16.

4. Cusanovich, D.A., Hill, A.J., Aghamirzaie, D., Daza, R.M., Pliner, H.A., Berletch, J.B., Filippova, G.N., Huang, X., Christiansen, L., DeWitt, W.S., et al. (2018). A Single-Cell Atlas of In Vivo Mammalian Chromatin Accessibility. Cell *174*, 1309–1324.e18.

Figure S1

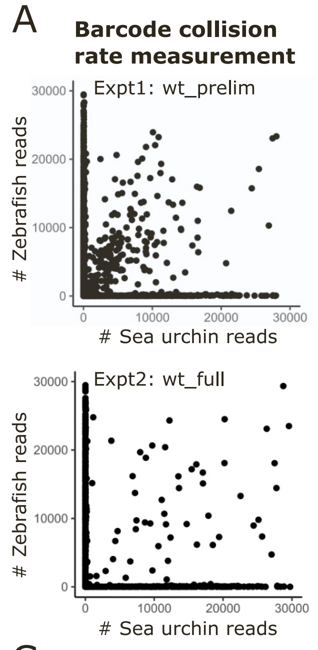

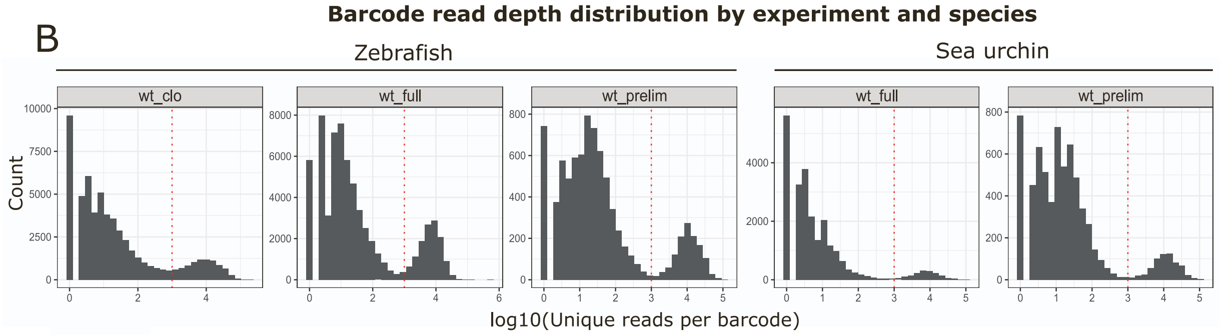

# Barcodes called as cells (Zebrafish)

Barcode selection threshold (# unique reads per barcode)	wt_clo	wt_full	wt_prelim	Total
None	59795	67078	8950	135823
1000	8580	12613	1246	22439
5000	5604	8226	1038	14868
10,000	3702	3873	669	8244

# Barcodes called as cells (Sea urchin)

Barcode selection threshold (# unique reads per barcode)	wt_full	wt_prelim	Total
None	29973	7618	37591
1000	1616	652	2268
5000	1121	545	1666
10,000	585	368	953

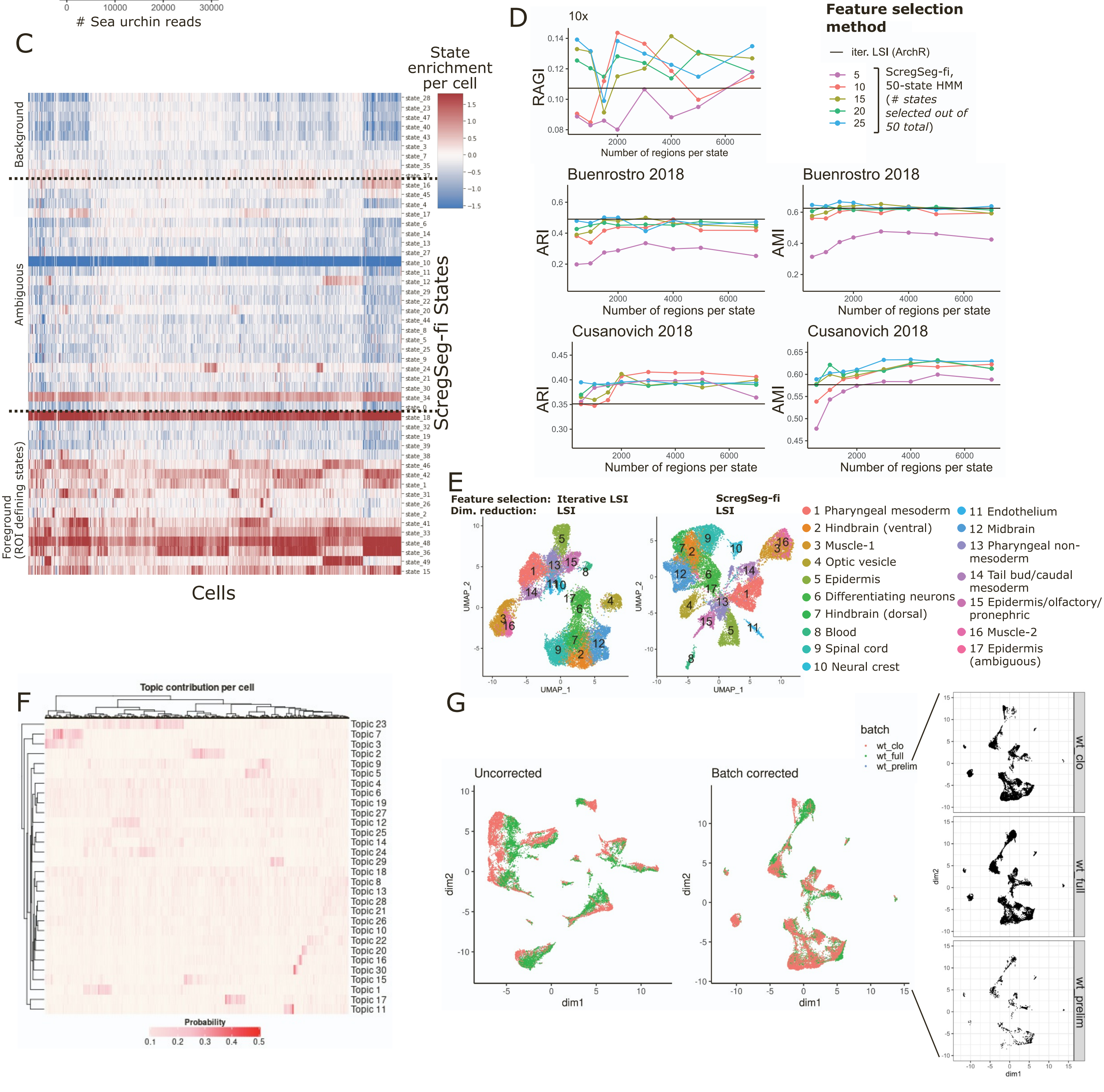

Figure S1 (related to Figure 1). Basic sci-ATAC-seq statistics and ScregSeg-fi benchmarking.

A. Assessment of the barcode collision events. Number of uniquely mapped reads per barcode that align to zebrafish (danrer11) and sea urchin (spur3.1) in the first and second experiments. Only barcodes with at least 1000 fragments in either species are considered.
B. Histogram of the number of uniquely mapped reads per barcode from alignment to zebrafish (danrer11) and sea urchin (spur3.1) in the two wild-type runs ("wt_full", "wt_prelim") and only zebrafish (danrer11) in the *npas4l*^{bns297} cloche mutant ("wt_clo"). Tables summarize the number of barcodes at different thresholds of reads per barcode.
C. State-cell association determined by the log-ratio between the observed state frequency (number of 1kb regions per state) within accessible sites of a given cell and the genome-wide state frequency (Fig 1C; see Methods). States are ordered by the state frequency (Figure 1C). Cells are ordered by hierarchical clustering (using seaborn.clustermap). High positive values represent a state enrichment in a given cell, thus revealing cell type specificity of that state.
D. Comparison of feature identification with Scregseg-fi and ArchR's iterative LSI on three different datasets: 10x Genomics PBMC data, hematopoiesis data (Buenrostro 2018) and mouse tissue cells (Cusanovich 2018). Residual average Gini-index (RAGI) was used to assess the clustering performance for 10x Genomics data, which measures the separation between known marker genes between clusters. Adjusted Rand index (ARI) and Adjusted mutual information (AMI) were used for the hematopoiesis and mouse tissue data, which compare the cluster identities against known cell labels. For all cases, we fitted a 50-state HMM. Subsequently, cell type identification was assessed using different numbers of putative informative states (using the 5, 10, 15, 20 and 25 rarest states) and different numbers of regions per state (using up to 500, 1000, 1500, 2000, 3000, 4000, 5000 and 7000 of the highest covered regions per state). The performance for cell type identification based on iterative LSI is depicted as a black horizontal line.
E. UMAP representation of 24hpf sciATAC-seq cells (this dataset) based on iterative LSI (using ArchR; left) or Scregseg-fi (right) derived (most variable) informative regions. Except for the feature selection step, processing was performed identically using the ArchR package (see Methods).
F. Heatmap representing the cell-topic probability from cisTopic.
G. UMAP representation of all filtered cells before and after batch correction, colored by batch. Batches are defined as the three sciATAC-seq experiment runs: two runs with wild-type embryos ("wt_full", "wt_prelim") and one run with *npas4l*$^{bns297/+}$, *npas4l*$^{+/+}$, *npas4l*$^{bns297/bns297}$ embryos ("wt_clo").

Figure S2

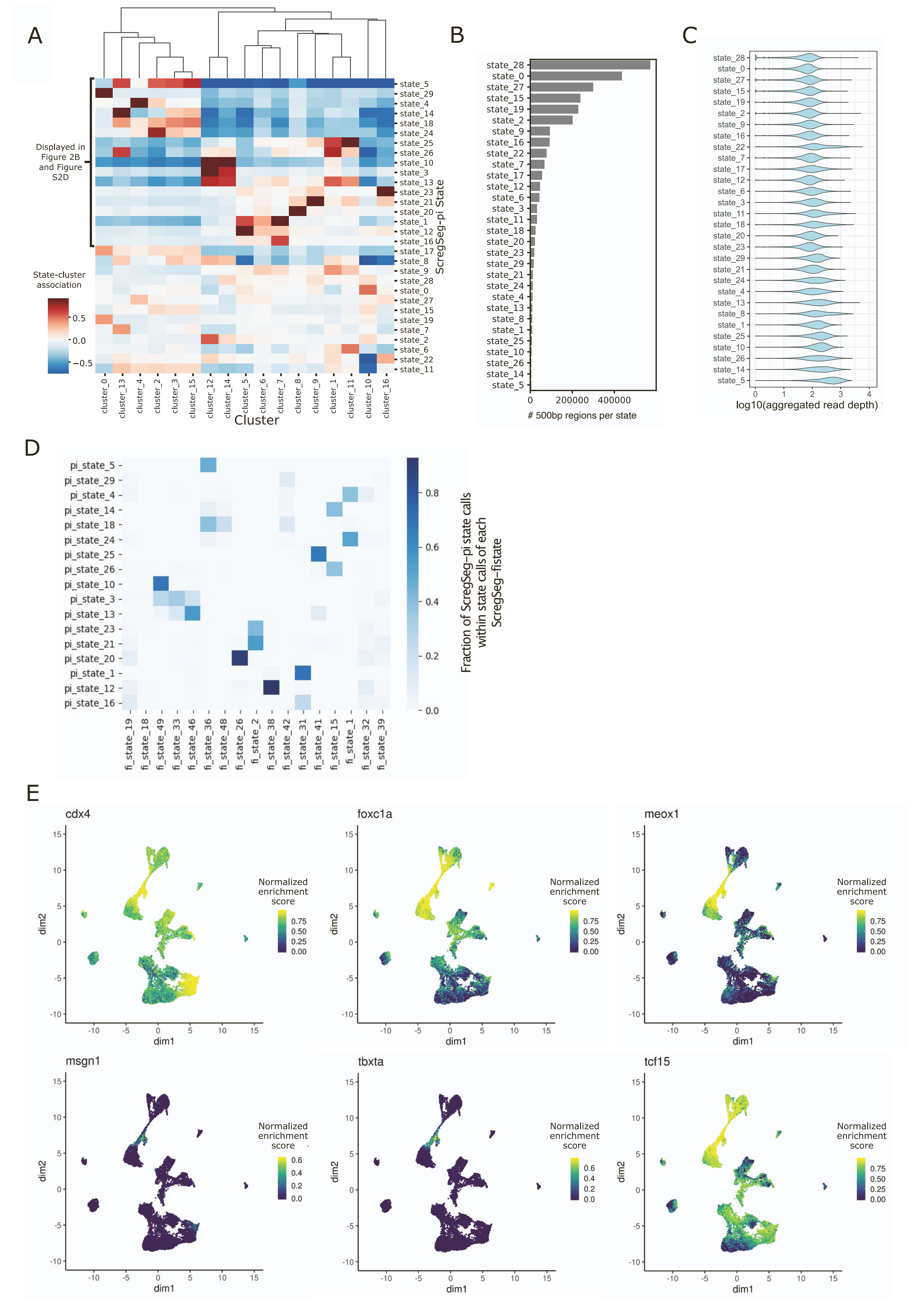

A
B
C
D
E
Displayed in Figure 2B and Figure S2D
State-cluster association
ScregSeg-pi State
Cluster
# 500bp regions per state
log10(aggregated read depth)
Fraction of ScregSeg-pi state calls within state calls of each ScregSeg-fistate
cdx4
foxc1a
meox1
msgn1
tbxta
tcf15
Normalized enrichment score
dim1
dim2

Figure S2 (related to Figure 2). Correspondence between ScregSeg-pi states and cell-type clusters or ScregSeg-fi states.

A. Heat map representing the association between each state with each cluster based on the log-fold enrichment between the ScregSeg-pi states' emission probabilities and the genomic background coverage profile (see Methods). Cells and states are ordered by hierarchical clustering (using seaborn.clustermap).
B. Number of 500 bp regions per state for Scregseg-pi.
C. Distribution of log-transformed read depth across all clusters per state for Scregseg-pi.
D. Correspondence between states of Scregseg-fi and Scregseg-pi. For Scregseg-fi and Scregseg-pi, the foreground states (Figure 1C) and the states from Figure 2B were selected, respectively. The overlap (correspondence) is determined by counting the joint state assignments across the genome for Scregseg-fi and Scregseg-pi and normalizing these counts by the number of regions assigned to each Scregseg-fi state.
E. Per cell distribution of accessibility at promoters of marker genes of the putative constituent cells of cluster14: neural mesodermal progenitors (*tbxta*, *cdx4*) and presomitic mesoderm (*foxc1a*, *meox1, msgn1*, *tcf15*), represented in UMAP space. Colour represents the rank-based AUCell enrichment score for a given region[36,114].

Figure S3

State	Motif	known TF
1	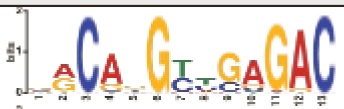	TP73
	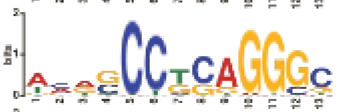	TFAP2C
3	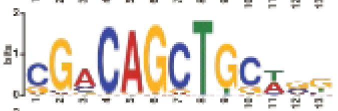	ASCL1
	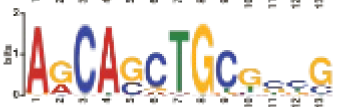	Myog
4	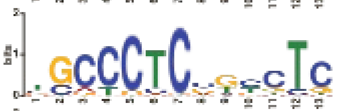	RARA:RXRG
5	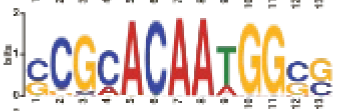	Sox2
10	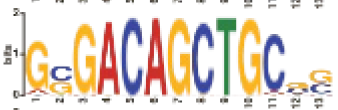	Myod1
12	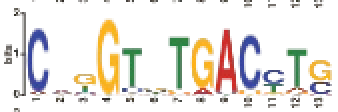	PAX5
	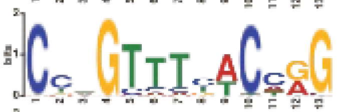	Unknown
	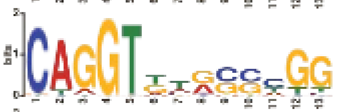	SNAI2
	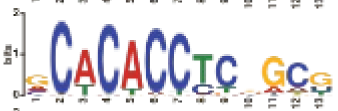	TBX15
13	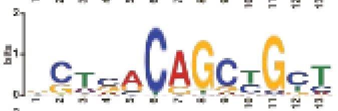	Myod1
	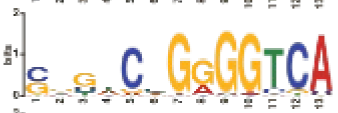	NR2C2
14	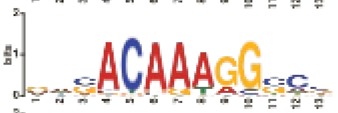	Sox3
	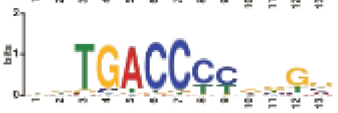	NR2F1

State	Motif	known TF
16	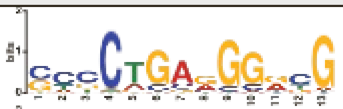	ZIC4
18	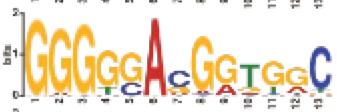	PPARG
20	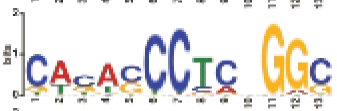	TFAP2B
	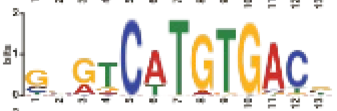	MITF
	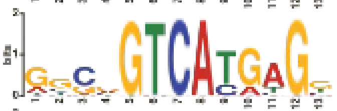	USF2
21	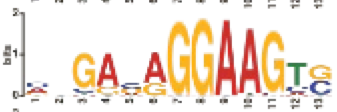	ETV6, FLI1
23	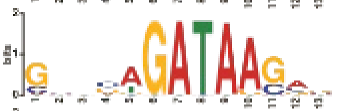	Gata4
24	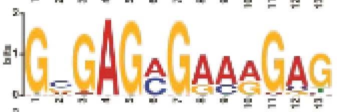	Unknown
25	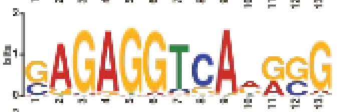	NR2F1
	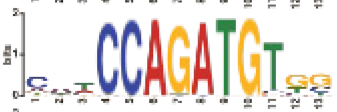	ZBTB18
26	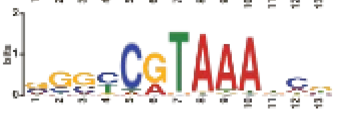	Hox11
29	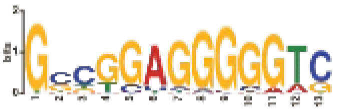	SP2

Figure S3 (related to Figure 3). Summary of motifs that are predictive of the Scregseg-pi states.

Figure S4

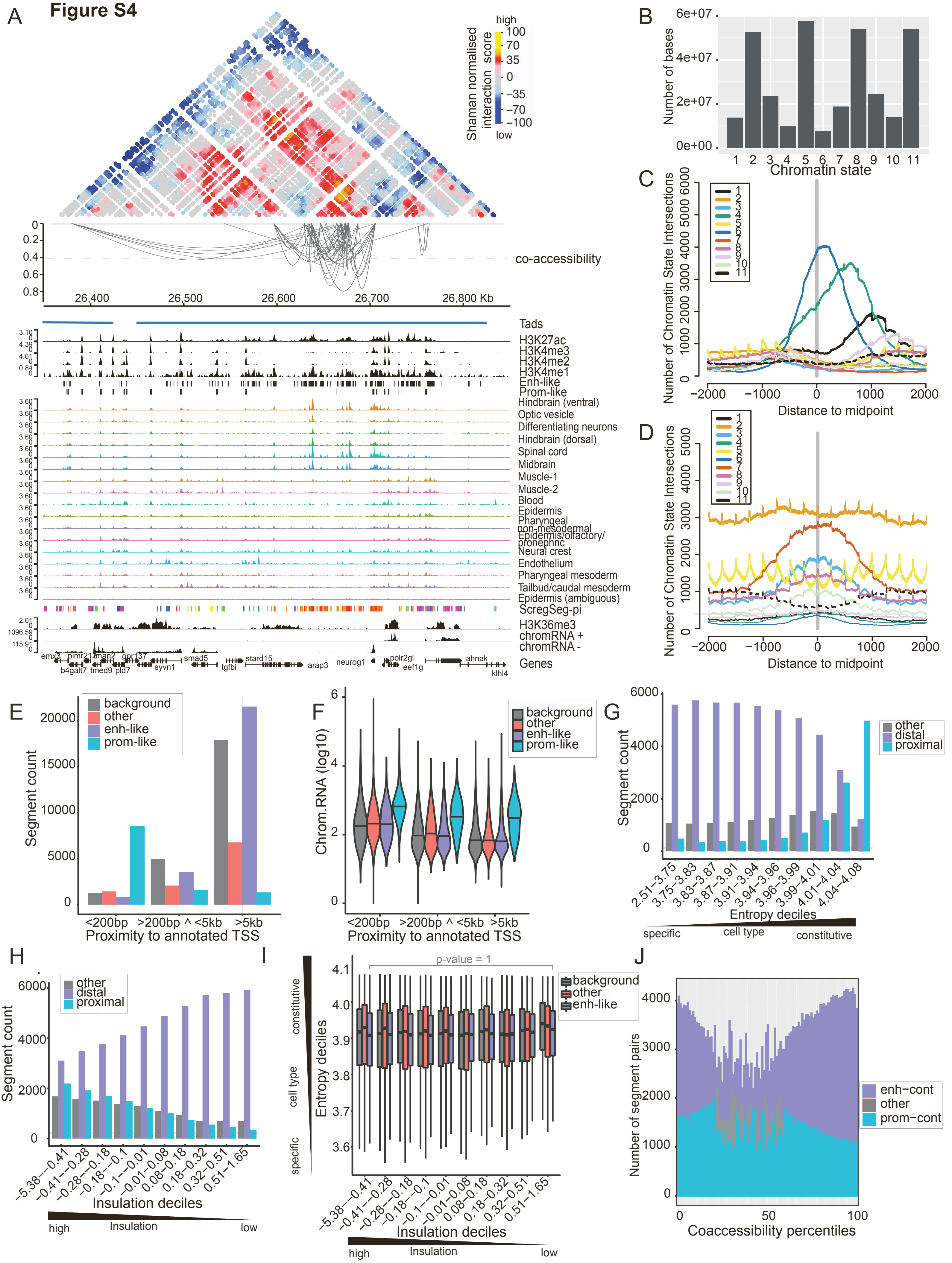
A
high
100
70
35
0
−35
−70
−100
low
Shaman normalised interaction score
co-accessibility
26,400
26,500
26,600
26,700
26,800 Kb
Tads
H3K27ac
H3K4me3
H3K4me2
H3K4me1
Enh-like
Prom-like
Hindbrain (ventral)
Optic vesicle
Differentiating neurons
Hindbrain (dorsal)
Spinal cord
Midbrain
Muscle-1
Muscle-2
Blood
Epidermis
Pharyngeal non-mesodermal
Epidermis/olfactory/pronephric
Neural crest
Endothelium
Pharyngeal mesoderm
Tailbud/caudal mesoderm
Epidermis (ambiguous)
ScregSeg-pi
H3K36me3
chromRNA +
chromRNA -
Genes
emx3
pimr212
man2
gpr137
b4galt7
tmed9
pld7
syvn1
smad5
tgfbi
stard15
arap3
neurog1
polr2gl
eef1g
ahnak
klhl4
B
Number of bases
Chromatin state
C
Number of Chromatin State Intersections
Distance to midpoint
D
Number of Chromatin State Intersections
Distance to midpoint
E
background
other
enh-like
prom-like
Segment count
<200bp
>200bp ^ <5kb
>5kb
Proximity to annotated TSS
F
Chrom.RNA (log10)
Proximity to annotated TSS
G
other
distal
proximal
Segment count
Entropy deciles
specific
cell type
constitutive
H
other
distal
proximal
Segment count
Insulation deciles
high
Insulation
low
I
p-value = 1
background
other
enh-like
Entropy deciles
constitutive
cell type
specific
Insulation deciles
high
Insulation
low
J
Number of segment pairs
enh−cont
other
prom−cont
Coaccessibility percentiles

Figure S4 (related to Figure 4). Characterization of hPTM states.

A. Browser shot as in Figure 4A except around the *neurog1* locus.
B. Number of bases genome-wide covered by each histone PTM state.
C. Histone PTM state coverage anchored on region midpoints that fall +/- 200 bp from annotated TSSs.
D. Histone PTM state coverage anchored on region midpoints that fall > 200 bp from annotated TSSs.
E. Number of foreground sci-ATAC-seq regions stratified by histone PTM classification types and distance to annotated TSSs.
F. Log10 chromatin RNA read counts for 5kb windows centered around intergenic foreground sci-ATAC-seq regions stratified by histone PTM classification types and distance to annotated TSSs.
G. Sci-ATAC-seq foreground region counts stratified by entropy score deciles and proximity to annotated TSS. “proximal” = <=200bp; ”other” = >200bp & <=5kb; “distal” = > 5kb.
H. Sci-ATAC-seq foreground region counts stratified by insulation score deciles and proximity to annotated TSS. “proximal” = <=200bp; ”other” = >200bp & <=5kb; “distal” = > 5kb.
I. *In situ* Hi-C insulation scores for foreground sci-ATAC-seq regions were split into deciles and then split according to their histone PTM type. The entropy score is plotted for the three non-promoter-like histone PTM types. P-value is the result of a Welch’s unpaired t-test for greater mean between insulation decile 1 and 10 for enhancer-like segments (mean 3.882884 vs 3.901317, df = 2169.2, n = 1400 and 3813).
J. Number of region pairs plotted for each stratification in Figure 4G. Region pairs are split first by Cicero co-accessibility score percentiles and then by having a promoter-like histone PTM state in one or both of the two regions (prom-cont), having no promoter-like histone PTM regions but having one or two enhancer-like PTM regions (enh-cont), or where neither region is promoter-like or enhancer-like (other).

Figure S5

A

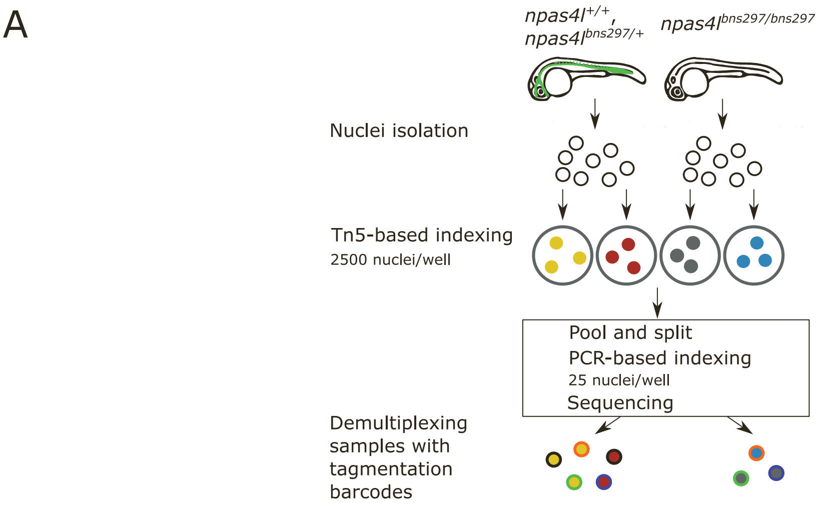

B

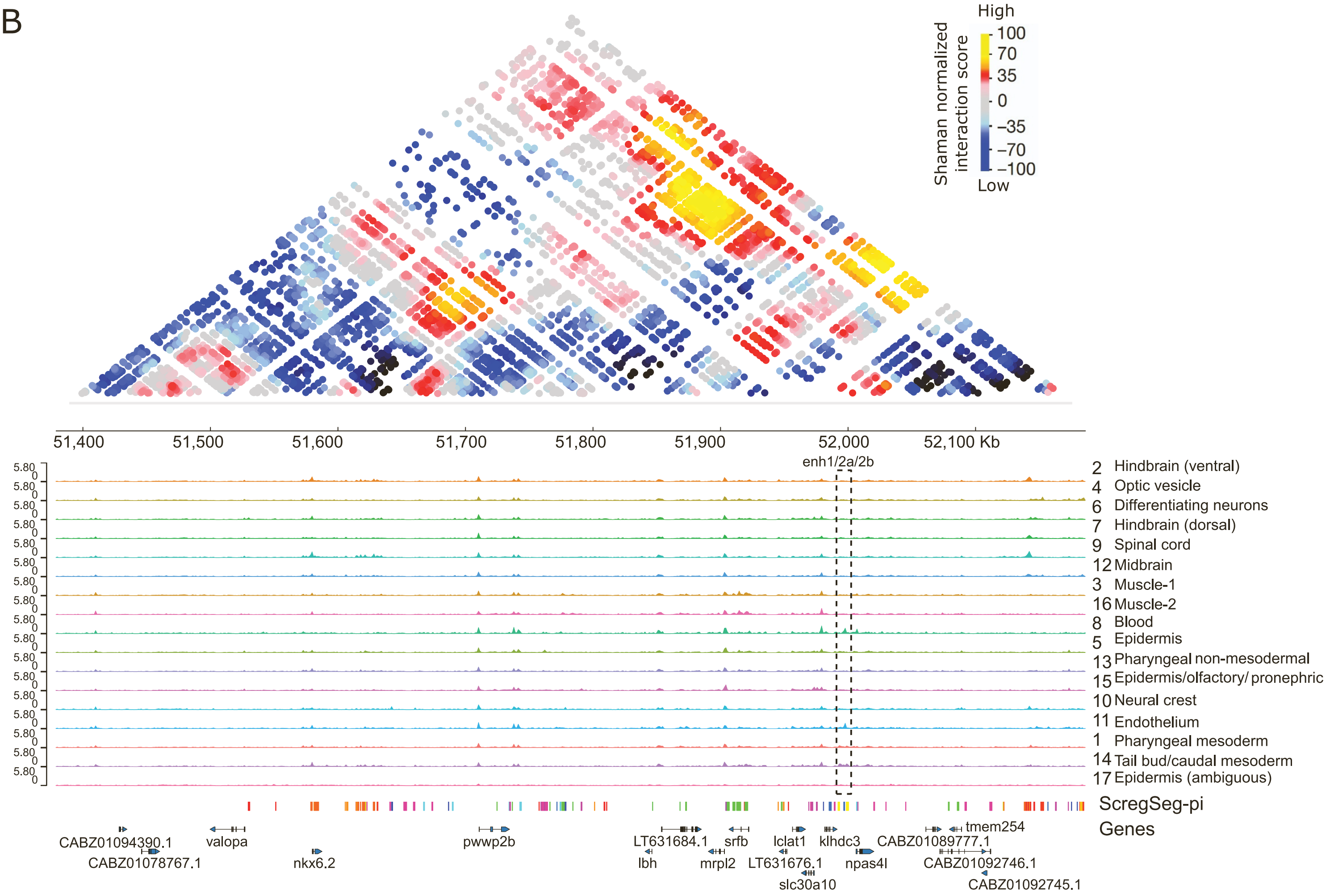

C — enh1

Motif ID	Match position	strand	q-value	Matched Sequence
KLF13	82-99	-	2.16E-06	ATGACACGCCCCCTTCTG
KLF14	85-98	-	5.58E-05	TGACACGCCCCCTT
KLF16	87-97	-	0.000214	GACACGCCCCC
SP8	86-97	-	0.000263	GACACGCCCCCT
SP4	84-100	-	0.000438	TATGACACGCCCCCTTC
HESX1	73-82	-	0.000651	GCTAATTGGC
LBX2	73-82	+	0.000664	GCCAATTAGC
GATA1::TAL1	228-245	-	0.000694	CTTATCTGTGCAGAACAC
EN2	73-82	+	0.00129	GCCAATTAGC
Gata1	237-247	-	0.00134	CTCTTATCTGT
GBX2	73-82	+	0.00138	GCCAATTAGC
GBX1	73-82	+	0.00219	GCCAATTAGC
SP3	87-97	-	0.00263	GACACGCCCCC
ESX1	73-82	+	0.00293	GCCAATTAGC
RAX	73-82	+	0.003	GCCAATTAGC
Gata4	236-246	-	0.00339	TCTTATCTGTG
GATA2	237-247	-	0.00399	CTCTTATCTGT
Barhl1	73-82	-	0.00432	GCTAATTGGC
GATA6	236-248	+	0.00607	CACAGATAAGAGC
CTCFL	82-95	+	0.00665	CAGAAGGGGGCGTG

D — enh2a

Motif ID	Match position	strand	q-value	Matched Sequence
RFX3	135-150	-	0.00365	CGCTGCTATGAAAACA
RFX2	135-150	-	0.004	CGCTGCTATGAAAACA
RFX5	135-150	-	0.00641	CGCTGCTATGAAAACA
POU6F2	57-66	-	0.00772	AGCTCATTAG
RFX3	135-150	+	0.00365	TGTTTTCATAGCAGCG
Rfx1	136-149	+	0.00782	GTTTTCATAGCAGC
EMX1	56-65	+	0.00802	CCTAATGAGC
RFX2	135-150	+	0.004	TGTTTTCATAGCAGCG
RFX4	135-150	-	0.00816	CGCTGCTATGAAAACA
SREBF2	60-69	+	0.00994	ATGAGCTGAT
BCL6B	82-98	+	0.0112	AGCTTTAGAGGATTCCA
TEAD2	100-112	+	0.0124	TGACATTCCCAGC
RFX5	135-150	+	0.00641	TGTTTTCATAGCAGCG
NOTO	56-65	-	0.0149	GCTCATTAGG
EVX2	56-65	-	0.0157	GCTCATTAGG
RFX4	135-150	+	0.00816	TGTTTTCATAGCAGCG
ZIC3	143-157	-	0.0168	ACTCCCCCGCTGCTA
FOXP2	133-143	-	0.018	ATGAAAACACA
HOXA5	24-31	-	0.0184	CACTAATG
SREBF1	60-69	-	0.019	ATCAGCTCAT

E — enh2b

Motif ID	Match position	strand	q-value	Matched Sequence
TFDP1	52-62	+	0.00047	CGGCGGGAAGG
Foxj3	57-73	+	0.00057	GGAAGGTAAATAAATAC
E2F4	52-62	+	0.000618	CGGCGGGAAGG
RXRG	12-25	+	0.00264	GGGTTCATAGGGCA
Hes2	23-35	-	0.00276	TAATGAAACGTGC
RXRB	12-25	+	0.00306	GGGTTCATAGGGCA
Rxra	12-25	+	0.00362	GGGTTCATAGGGCA
ZIC3	144-158	+	0.00346	GACCTCCTGCTGCAA
Nr2f6(var.2)	11-25	+	0.00381	GGGGTTCATAGGGCA
Nkx3-1	5-13	-	0.00404	CCCACTTAA
NKX3-2	5-13	-	0.00423	CCCACTTAA
NKX2-3	4-13	-	0.00418	CCCACTTAAA
Nr2f6	12-25	+	0.00568	GGGTTCATAGGGCA
FOXC2	59-70	+	0.00579	AAGGTAAATAAA
FOXF2	57-70	+	0.00802	GGAAGGTAAATAAA
GATA1::TAL1	94-111	-	0.0084	TTTATCAGAGCGCTACAT
E2F6	52-62	+	0.0108	CGGCGGGAAGG
Hoxd3	26-41	-	0.0127	CAGAGTTAATGAAACG
FOXC1	59-69	+	0.0136	AAGGTAAATAA
HEY2	22-31	-	0.0147	GAAACGTGCC

Figure S5 (related to Figure 5). *Npas4l* mutant sci-ATAC-seq and examination of putative enhancers of *npas4l*.

A. Schematic of sci-ATAC-seq data collection from *npas4l* mutant embryos. The Tg(fli1a:nls-GFP)^{y7} background was employed to separate homozygous cloche mutant (*npas4l*$^{bns297/bns297}$) embryos from heterozygous and homozygous wild type siblings (*npas4l*$^{bns297/+}$, *npas4l*$^{+/+}$) based on the loss of a fli1a-GFP+ endothelial cells in the mutant. These two pools of embryos were kept separate for the nuclei isolation and first indexing step (tagmentation). All nuclei were pooled for subsequent steps (as figure 1A) and homozygous mutant vs sibling cells could be distinguished based on their tagmentation barcodes.
B. SHAMAN interaction scores surrounding the *npas4l* locus and it's putative enhancers enh1, enh2a, enh2b.
C. The top 20 motif matches within enh1 as detected by FIMO[112] using the JASPAR motif database[68].
D. The top 20 motif matches within enh2a.
E. The top 20 motif matches within enh2b.
